# Generation and Improvement of Effector Function of a Novel Broadly Reactive and Protective Monoclonal Antibody against Pneumococcal Surface Protein A of *Streptococcus pneumoniae*

**DOI:** 10.1371/journal.pone.0154616

**Published:** 2016-05-12

**Authors:** Sascha A. Kristian, Takayuki Ota, Sarah S. Bubeck, Rebecca Cho, Brian C. Groff, Tsuguo Kubota, Giuseppe Destito, John Laudenslager, Lilia Koriazova, Tomoyuki Tahara, Yutaka Kanda

**Affiliations:** 1 Kyowa Kirin Pharmaceutical Research, Inc., 9420 Athena Circle, La Jolla, CA 92037, United States of America; 2 Kyowa Hakko Kirin Co., Ltd., R&D Division, 1-6-1, Ōtemachi, Chiyoda-ku, Tokyo 100–8185, Japan; New York State Dept. Health, UNITED STATES

## Abstract

A proof-of-concept study evaluating the potential of *Streptococcus pneumoniae* Pneumococcal Surface Protein A (PspA) as a passive immunization target was conducted. We describe the generation and isolation of several broadly reactive mouse anti-PspA monoclonal antibodies (mAbs). MAb 140H1 displayed (i) 98% strain coverage, (ii) activity in complement deposition and opsonophagocytic killing (OPK) assays, which are thought to predict the *in vivo* efficacy of anti-pneumococcal mAbs, (iii) efficacy in mouse sepsis models both alone and in combination with standard-of-care antibiotics, and (iv) therapeutic activity in a mouse pneumonia model. Moreover, we demonstrate that antibody engineering can significantly enhance anti-PspA mAb effector function. We believe that PspA has promising potential as a target for the therapy of invasive pneumococcal disease by mAbs, which could be used alone or in conjunction with standard-of-care antibiotics.

## Introduction

*S*. *pneumoniae* is a major cause of severe bacterial infections including pneumonia, bacteremia and meninigitis as well as less serious but prevalent diseases such as acute and chronic otitis media [1].

Capsular polysaccharide based vaccines are effective in reducing the incidence of invasive pneumococcal disease [2]. However, *S*. *pneumoniae* remains a substantial cause of morbidity and mortality in the US and elsewhere [1, 3] and the emergence of non-vaccine and multi-drug resistant pneumococcal strains is of concern [4, 5]. Thus, development of alternative or adjunctive anti-*S*. *pneumoniae* drugs is needed. Passive antibody therapy may represent an approach to complement antibiotic treatment as well as existing and new-generation vaccines.

PspA is a well-characterized pneumococcal virulence factor [6] with potential as a vaccine candidate [2]. A PspA vaccine has been used in a clinical trial [7] and new PspA vaccines are currently in the pre-clinical stage [2]. However, the highly variable nature of PspA makes it a challenging vaccine candidate. PspA is classified into 3 families with 6 clades based on sequence similarities in the variable N-terminal α-helical region [8]. PspA also contains a more conserved proline-rich region, which may include a non-proline block, and a choline-binding domain [8]. With the vast majority of clinical *S*. *pneumoniae* isolates being family 1 or 2 PspA (Clades 1–5) [9], vaccines and antibody drugs should cover these PspA types to be broadly protective.

We speculate that developing a passive immunization drug containing anti-PspA mAbs has great potential as (1) it is believed that PspA is expressed in all pneumococcal strains implying a broad, serotype-independent protection and (2) portions of PspA extrude from the pneumococcal capsule giving such mAbs the potential to be opsonic and confer complement deposition and phagocytosis of the bacteria [10].

In this paper, the generation of a broadly reactive and protective anti-PspA mAb, 140csH1, with potential for use in the treatment of invasive pneumococcal infections is described. Moreover, we show that anti-PspA mAb effector function could be significantly enhanced by mAb engineering.

## Materials and Methods

### Bacterial strains, culture conditions, preparation of labeled pneumococci

The bacterial strains used in this study are listed in Table 1. Bacteria were grown to exponential phase in Todd Hewitt Broth (Becton Dickinson), 0.5% yeast extract (THY) at 37°C with 5% CO_2_. For most assays, bacteria were washed twice with sterile, endotoxin free phosphate buffered saline, pH 7.4 (PBS; Invitrogen) and adjusted photometrically to the desired colony forming unit (CFU) concentrations which were confirmed by plating serial dilutions onto Columbia agar, 5% sheep blood plates (SBA plates; Biomérieux). To label *S*. *pneumoniae* D39 with fluorescein for phagocytosis assays, exponential phase cells in PBS were heat-inactivated for 60 min at 56°C, washed twice, adjusted to 10^9^ bacterial particles/mL in PBS with 100 μg/mL FITC (Invitrogen) and incubated overnight at 4°C with rotation. The next morning, bacteria were washed 5x with PBS and aliquots were made and stored at -80°C.

**Table 1:** *S*. *pneumoniae* strains used in this study.

#	Strain	Serotype	PspA Family	PspA Clade	Source
1	ATCC-6301	1	1	1	ATCC
2	NCTC-11910	23F	1	1	NCTC
3	ATCC-49136	17	1	1	ATCC
4	ATCC-49619	19F	1	1	ATCC
5	NCTC-11888	6B	1	1	NCTC
6	EF3030	19	1	1	CDC
7	BAA-658	6B	1	1	ATCC
8	ATCC-700675	6B	1	1	ATCC
9	ATCC-6305	5	1	2	ATCC
10	WU2	3	1	2	CDC
11	NCTC-7978	3	1	2	NCTC
12	NCTC-7466 (D39)	2	1	2	NCTC
13	NCTC-11886	4	2	3	NCTC
14	BAA-475	19A	2	3	ATCC
15	BAA-340	14	2	3	ATCC
16	ATCC-700905	19F	2	3	ATCC
17	PJ-1324	6B	2	3	Intercell
18	BAA-334 (TIGR4)	4	2	3	ATCC
19	NCTC-11902	14	2	4	NCTC
20	NCTC-11905	18C	2	4	NCTC
21	NCTC-11906	19F	2	4	NCTC
22	ATCC-700673	19A	2	5	ATCC
23	ATCC-6303	3	2	5	ATCC
24	NCTC-11897	9V	2	5	NCTC
25	BAA-612	6B	2	5	ATCC
26	ATCC-49150	?	?	?	ATCC
27	ATCC-700671	9V	?	?	ATCC
28	DS2341-94	4	?	?	CDC
29	D39 PspA mutant	2	1	2	This study.
30	PJ-1324 PspA mutant	6B	2	3	Intercell
31	TIGR4 PspA mutant	4	2	3	Intercell
32	BG8743	23	1	1	UAB
33	AC094	9L	1	1	UAB
34	BG6692	33	1	1	UAB
35	BG8838	6	1	1	UAB
36	DBL1	6B	1	1	UAB
37	BG9739	4	1	1	UAB
38	DBL6A	6A	1	1	UAB
39	L81905	4	1	1	UAB
40	DBL5	5	1	2	UAB
41	E134	23F	1	2	UAB
42	EF10197	3	1	2	UAB
43	EF6796	6A	1	2	UAB
44	BG9163	6B	1	2	UAB
45	AC122	9V	2	3	UAB
46	BG8090	19	2	3	UAB
47	BG7561	15	2	4	UAB
48	BG7817	12	2	4	UAB
49	BG11703	18	2	4	UAB
50	EF5668	4	2	4	UAB
51	BG6380	37	3	6	UAB

ATCC, American Type Culture Collection (Manassas, VA)

NCTC, National Collection of Type Cultures (Salisbury, UK)

Intercell, Intercell AG (Vienna, Austria)

CDC, Centers for Disease Control and Prevention (Atlanta, GA)

UAB, University of Alabama at Birmingham.

### Mice

All animal study procedures were reviewed and approved by the Institutional Animal Care and Use Committee at La Jolla Institute for Allergy and Immunology (LJI). Female CD-1 and Swiss Webster mice were purchased from Taconic Farms and Charles River Laboratories International, respectively. Euthanization of mice was carried out using carbon dioxide asphyxiation followed by cervical dislocation as a confirmation.

### Generation of PspA deficient *S*. *pneumoniae* mutants

Generation of the TIGR4 and PJ-1324 PspA negative mutant strains was previously described [11]. Precise, in-frame allelic replacement of the *S*. *pneumoniae* D39 *pspA* gene with a chloramphenicol (Cm) resistance gene (*cat*) was performed using published D39 genome sequence information [12] and plasmid pC194 isolated from *Staphylococcus aureus* RN2887 (NRS131) as template for cloning the *cat* gene by PCR. Strain NRS131 was obtained from the Network on Antimicrobial Resistance in *Staphylococcus aureus* (NARSA; Chantilly, Virginia). Briefly, PCRs and genomic D39 DNA were used to amplify ~1,000-bp sequences immediately upstream and downstream of *pspA*. Primers were constructed with 20-base pair 5' extensions corresponding to the 5' and 3' ends of the *cat* gene. In a fusion PCR, the upstream and downstream PCR products were then combined with an amplicon of the complete *cat* gene. The fusion PCR product was then cloned into pCR^®^-Blunt II-TOPO^®^ vector (Invitrogen) and *Escherichia coli* DH5α cells transformed and selected on agar plates containing 10 μg/mL Cm. In-frame substitution of *pspA* with *cat* in plasmids isolated from Cm-resistant *E*. *coli* clones was verified by sequencing. A linear knock out construct was PCR amplified from plasmid preparations and purified. *S*. *pneumoniae* D39 cells were then transformed in the presence of competence stimulating peptide-1 [13] and transformants selected on SBA plates containing Cm. Deletion of *pspA* in Cm-resistant transformants was confirmed by PCR and sequencing of genomic DNA. Finally, the absence of PspA in mutant cells on the protein level was verified by lack of anti-PspA antibody binding in flow cytometry experiments.

### Antigen preparation for mouse immunizations

Recombinant, truncated PspA proteins derived from *S*. *pneumoniae* strains D39, TIGR4, ATCC-700675, and BAA-612 were prepared. The PspA proteins encompassed the N-terminal α-helical coiled-coil and proline-rich regions, but not the choline-binding domains. The described PspA fragment was cloned from each respective strain into pET20b(+) (Novagen) using standard cloning methods. The final constructs included a 22 amino acid signal peptide sequence from pectate lyase B (pelB) at the N-terminus and a C-terminal 6xHis tag. The completed expression vectors were transformed into DH5α-T1R *E*. *coli* (Invitrogen) and plasmid DNA isolated from ampicillin-resistant cultures. Sequence was confirmed by Sanger DNA sequencing (Genewiz, Inc.). Next, plasmid DNA for each of the PspA constructs was transformed into chemically competent *E*. *coli* BL21(DE3) (Stratagene) and plasmid-containing clones were selected after growth on ampicillin LB-Miller agar plates. PspA expression was induced by addition of isopropyl β-D-1-thiogalactopyranoside (IPTG) (BioPioneer Inc.) to the culture medium, bacteria harvested, lysed (microfluidizer) and lysate sterilized using a 0.22-μm filter unit (Millipore). Filtered lysate was then purified by metal chelate affinity chromatography with Ni Sepharose 6 Fast Flow resin (GE Healthcare) according to the manufacturer instructions. PspA was eluted from the column with 200 mM imidazole, and subsequently dialyzed against 10 mM Tris-HCl buffer, pH 7.5, 0.1M NaCl. Protein concentration was determined by DC Lowry protein assay (Bio-Rad) using BSA standard (Pierce Biotechnology) in the same buffer.

### Mouse immunizations, titer testing, hybridoma generation, anti-PspA mAb isolation, isotyping, and purification

Two immunization protocols with female BALB/c mice were applied during this study. Protocol 1 (most animals): mice were given three intraperitoneal injections (2 week intervals) of D39 or TIGR4 PspA proteins (20 μg dose in 200 μL Sigma Adjuvant System^®^, Sigma-Aldrich). 2–9 weeks after the third injection, mice received one final intravenous boost of the respective PspA antigen (20 μg, no adjuvant). Protocol 2: Mice received priming and two booster injections with 20 μg of D39 or TIGR4 PspA proteins as described above. After the third immunization, the mice were allowed to rest for around 90 days then given a 4^th^ immunization. Three weeks later, the D39 and TIGR4 PspA immunized mice received a final booster injection with 20 μg of recombinant, truncated PspA from *S*. *pneumoniae* ATCC-700675 or BAA-612, respectively. Three days after the final booster injection, mice were euthanized and the spleens collected and homogenized. Splenocytes from hyper-immunized mice were used as fusion partners with Sp2/0-Ag14 mouse myeloma cells (American Type Culture Collection #: CRL-1581) for production of hybridomas. Fusions were conducted according to standard methods. After fusion, hybridoma supernatants were tested for the presence of anti-PspA IgG-type antibodies. During primary screenings, hybridoma supernatants were tested for IgG binding to the recombinant protein antigens by ELISA and to a limited panel of *S*. *pneumoniae* strains by flow cytometry. Subsequently, single hybridoma clones secreting anti-PspA mAbs were obtained through limited dilution technique according to standard methodology and re-tested as above. To identify the isotype of isolated mouse mAbs, IsoQuick Strips^™^ (Sigma-Aldrich) were used.

### Generation of overlapping recombinant PspA fragments and ELISA for epitope mapping

Overlapping MBP-tagged recombinant PspA fragments, each containing an identical N-terminal MBP tag, were expressed in *E*. *coli* BL21(DE3) (Invitrogen). Individual plasmids containing either a control non-PspA fusion peptide (MBP-B6R) or one of four overlapping PspA fragments from *S*. *pneumoniae* strain BAA-658 were generated. *E*. *coli* were transformed with plasmids and plated on ampicillin-containing LB-Miller agar plates. Individual colonies were subsequently cultured in LB-Miller broth and induced to express recombinant peptides with 1mM IPTG for 3 hours. Bacteria were collected by centrifugation and the pellet was resuspended in 50 μL PBS followed by three freeze-thaw cycles (1 min ethanol-dry ice bath, 1 min 37°C water bath followed by vortexing) to promote lysis. Lysate was cleared of insoluble debris by adding 1.2 mL PBS followed by centrifugation.

NUNC MaxiSorp^™^ ELISA plates were coated overnight with 50 μL cleared bacterial lysate (4°C) then blocked with 200 μL per well SuperBlock^®^ (TBS) Dry Blend blocking buffer (Thermo Scientific) for 30 min at room temperature. Lead anti-PspA antibodies 139G3, 140G1, 140H1, 140G11 and rabbit-anti-MBP (New England Biolabs) were diluted in 1x TBST (0.05% Tween-20 in Tris Buffered Saline, pH 8 (Sigma)) to 1 μg/mL. 50 μL of diluted mAb was added to respective antigen coated wells in duplicate, and incubated for 1h at 37°C. 50 μL HRP-conjugated donkey-anti-mouse IgG or HRP-conjugated goat-anti-rabbit IgG (Jackson Immunoresearch), diluted 1:5,000 in 1% SuperBlock^®^/TBST was added to appropriate wells. After 1h incubation at 37°C HRP-conjugated secondary antibodies were developed with 100 μL peroxidase-reactive colorimetric substrate (TMB + Substrate-chromogen (DAKO)). Colorimetric reaction was stopped with 50 μL 2.0 N sulfuric acid (LabChem, Inc.) after 5–10 min. The absorbances of individual ELISA wells were measured at 450 nm (A450) using a VersaMax microplate reader (Molecular Devices). Wells with higher A450 values were considered to contain primary antibodies with affinity for the PspA protein coated in that well.

### Affinity measurements

In order to kinetically analyze the binding activity of anti-PspA mouse antibodies 139G3, 140G1, 140G11 and 140H1, the binding activity was measured by surface plasmon resonance method (SPR). Three different recombinant PspA proteins (PspA-D39, PspA-BAA-658 and PspA-TIGR4) were immobilized on different CM5 sensor chips (GE Healthcare Bio-Sciences) using an amine coupling method. Anti-PspA antibodies were then diluted in two-fold serial dilutions (40 nM to 1.25 nM or 20 nM to 0.625 nM concentration ranges) and added to the coated sensor chips at a flow rate of 30 μL/min using a Biacore 3000 (GE Healthcare Bio-Sciences). Bivalent binding modeling was used to analyze the binding of each mAb (Biacore 3000 Evaluation software, Biacore). As a result, an association rate constant ka1, a dissociation rate constant kd1, and a dissociation constant K_D1_ (kd/ka), as well as an association rate constant ka2, a dissociation rate constant kd2, and a dissociation constant K_D2_ (kd/ka), for each individual antibody were obtained.

### Isotype switching of mouse anti-PspA antibodies from IgG1 to IgG2a

Vectors for the expression of “class-switched” recombinant mouse IgG2a, kappa-isotype versions of antibodies 140G1 or 140H1 (140csG1, 140csH1) were created by in-frame fusions of gene fragments of each antibody clone’s variable region heavy chain domain (VH) to a gene fragment coding for mouse IgG2a constant heavy chain (CH) immunoglobulin. The in-frame fusions were generated using standard methodology and transformed into DH5α-T1R chemically competent *E*. *coli* (Invitrogen). Plasmid DNA was isolated from colonies selected on ampicillin-containing LB-Miller agar plates, screened by restriction digest and confirmed by Sanger-sequencing (performed by GENEWIZ, Inc.) for each of expression vector containing the class-switched antibody heavy chains. For each antibody, the mouse kappa light chain was amplified by standard PCR methods from a plasmid template containing the desired kappa chain. The kappa chain gene cDNAs were inserted into the digested expression vectors and the ligation mixture transformed into DH5α-T1R chemically competent *E*. *coli* as described previously. A plasmid for each class-switched antibody expression vector was prepared from a selected clone of the transformant that was confirmed by restriction digest and finally Sanger-sequencing (performed by GENEWIZ, Inc.). As a result, mammalian expression vector plasmids for expression of class-switched antibodies 140csG1 or 140csH1 containing mouse IgG2a heavy chains and mouse kappa chains were generated. Flow cytometry experiments with several *S*. *pneumoniae* strains demonstrated the binding of 140csG1 and 140csH1 was comparable to parental mAbs indicating no loss of affinity through isotype-switching (data not shown).

### Preparation of mouse/human chimeric anti-PspA antibodies

The mouse/human chimeric antibodies (human IgG1, kappa) from 140G1 and 140H1 were prepared following the method of Shitara *et al*. [14]. The engineered IgG1/IgG3 Fc is the previously described 113F variant [15] with a single amino acid substitution N392K (EU numbering). All Fu+ and Fu- antibody variants were produced using transiently transfected CHO cell lines as previously described [15, 16] and purified using standard antibody purification techniques.

### Absorption of human plasma for complement deposition assays (CDAs)

To remove anti-pneumococcal antibodies from human plasma used for CDAs, fresh plasma isolated from heparinized blood obtained from a healthy volunteer was absorbed with *S*. *pneumoniae* PJ-1324 cells. Briefly, exponential phase pneumococci were washed twice in PBS, then pelleted and resuspended in plasma at a concentration of ~2x10^10^ CFU/mL. The bacterial suspension was rotated overhead on ice for 60 min. Then, bacteria were pelleted, the plasma supernatant removed and subjected to a second round of absorption as above. Then, bacteria were removed by centrifugation, the twice absorbed plasma sterile filtered and stored in aliquots at -80°C until use.

### Culture and differentiation of HL-60 cells

HL-60 (ATCC#: CCL-240^™^) cells were differentiated into polymorphonuclear neutrophil (PMN)-like cells as previously described [17]. Briefly, HL-60 cells were cultured at 4–5x10^5^/mL in differentiation medium (80% RPMI-1640 (Invitrogen), 10 mM L-glutamine, 20% fetal bovine serum (FBS; Hyclone Laboratory) and 100 mM *N*, *N*-dimethylformamide (DMF; Sigma-Aldrich)) for 4–7 days at 37°C, 5% CO_2_. Cell viability exceeded 90–95% as assessed by trypan blue exclusion. CD11b, CD35, and CD71 expression was assessed by flow cytometry to verify successful differentiation.

### Isolation of human blood PMN

PMN were isolated from heparinized human blood obtained from healthy donors recruited through La Jolla Institute for Allergy and Immunology (LJI) normal donor blood donation program which was approved by the LJI IRB and is in compliance with all regulations and policies. Written informed consent for blood donation is given by the donors. Briefly, whole blood was sedimented in dextran to remove the red blood cells, and PMNs were isolated by density centrifugation in Ficoll-Paque^™^ Plus (GE Healthcare Bio-Sciences AB). Sedimented PMNs were collected and erythrocytes hypotonically lysed with endotoxin-free distilled water (Invitrogen). Viability of the cells exceeded 95% as assessed by trypan blue exclusion.

### Antibody binding to live pneumococci

Anti-PspA antibody binding to live, exponential phase pneumococci was determined by flow cytometry. Briefly, bacteria were washed in PBS then co-incubated with mouse hybridoma supernatants or purified anti-PspA mAbs. Mouse, human or chimeric isotype primary antibodies or secondary antibodies alone served as negative controls. After washing the cells, bound primary antibody was detected with PE-labeled anti-mouse (goat IgG, polyclonal) or anti-human (goat F(ab')_2_ IgG, polyclonal) IgG antibodies (SouthernBiotech, #1030–09, #2043–09, respectively). The FL-2 fluorescence intensity of bacterial particles was measured with a FACSCalibur^™^. Forward and side scatters were adjusted with logarithmic signal amplification using a gate set on unstained bacteria. FL-2 mean fluorescence intensities (MFI) of ≥ 10,000 bacterial particles per sample were measured after excitation with a 488 nm laser.

### CDAs with mouse serum, human serum or absorbed human plasma

**~**10^8^ pneumococcal CFU were incubated for 30 min in polypropylene 96-well round bottom plates (Corning Inc.) in 0.2 mL Hanks’ Balanced Salt Solution (HBSS), 3.75% bovine serum albumin (BSA; Sigma-Aldrich), 37°C, 600 rpm horizontal shaking with or without mouse or chimeric isotype or anti-PspA mAbs and commercially available Balb/c or CD-1 mouse serum (Innovative Research), normal human serum pool (Quidel Corp.) or absorbed human plasma at concentrations optimized for each bacterial strain. Cells were then washed with ice-cold PBS, 0.5% BSA, and resuspended in 100 μL refrigerated PBS, 0.5% BSA, containing 2 μg/mL fluorescein-labeled anti-mouse or anti-human C3 antibodies (Cedarlane Laboratories USA Inc.). After 30–60 min incubation at 4°C, bacteria were washed in ice-cold PBS, 0.5% BSA. After a final washing step, the cells were analyzed by flow cytometry; the FL-1 intensity of ≥ 20,000 gated bacterial particles per sample was measured and recorded.

### Opsonophagocytic killing (OPK) assays with PMN-like HL-60 cells

~2x10^4^ CFU of PJ-1324 were opsonized for 60 min, 4°C, 750 rpm in 100 μL of 85% HBSS/0.1% BSA, 5% PBS, 10% baby rabbit serum (AbD Serotec) in polypropylene 96-well round bottom plates with 1 μg/mL mouse IgG2a isotype control mAb C44 purified from a hybridoma cell line (ATCC#: CRL-1943^™^) or mouse anti-PspA mAbs. In some experiments, heat-inactivated serum was used to evaluate the significance of active complement for OPK activity. 10^6^ differentiated PMN-like HL-60 cells in HBSS, 10 mM glucose were added to the bacteria. As a control, bacteria were also incubated under the same conditions without phagocytes. Plates were incubated at 37°C with mixing (750 rpm). At various time points, CFU were determined by serially diluting the samples in sterile distilled water to hypotonically lyse PMNs and plating on THY agar plates.

### Opsonophagocytosis assays (OPAs) with human PMN and chimeric anti-PspA antibodies

In 96-well polypropylene round bottom plates, 2.5x10^6^ fluorescein-labeled pneumococcal particles were incubated for 5–30 min with 5x10^5^ purified human PMN in 200 μL HBSS, 10% PBS containing 0–10 μg/mL isotype or chimeric anti-PspA antibody. Samples were washed once and ethidium bromide (0.25 mg/mL final) was added to the samples to differentiate between intra- and extracellular pneumococci. Samples were typically run in quadruplicate and 200 live PMN/sample analyzed for phagocytosis of pneumococci.

### Mouse passive immunization experiments

Exponential phase bacteria were washed twice in PBS and either injected i.p. or intravenously (i.v.) into unanesthetized mice, or intranasally (i.n.) or intratracheally (i.t.) into mice anesthetized by 2, 2, 2-Tribromoethanol (Sigma Aldrich) in PBS. For the i.n. instillation, 40 μL bacterial suspensions were dropped into the nostrils; for i.t. instillation, 50 μL bacteria were delivered through aspiration by occluding the trachea of the mice with the ball of a sterile animal feeding needle (20 G x 1-1/2“; Cadence Science) using a Hamilton 100-μl syringe (Hamilton Company). In prophylactic settings, 100–300 μg isotype control or anti-PspA mAbs in 100 μL PBS were given i.p. 4–6 h before infection. In therapeutic settings, mAbs were administered in 200 μL PBS within 24 h post-infection. In some experiments, the β-lactam antibiotic ceftriaxone or the macrolide erythromycin (ERM) (both Sigma-Aldrich) were used alone or in combination with mAbs, injected either i.p. or i.v. in 100–200 μL PBS or intragastrically (i.g.) within 1 h post-infection. For i.g. administration of 200 μL PBS, 1.5% ethanol (Pharmaco-Aaper) ± 1.5 mg/mL cell-culture tested ERM, sterile animal feeding needles (20 G x 1-1/2“; Cadence Science) were used.

In most experiments, 20 μL heparinized tail vein blood was collected after infection to enumerate bacterial CFU. In some pneumonia experiments, lungs were collected 24 h after infection and homogenized in tubes containing 1-mm Zirconia/Silica beads (BioSpec Products) and ice-cold PBS. After collection, organ weights were determined and organs were homogenized with a Minibeadbeater^™^ (BioSpec Products). For both blood and organ homogenates, 10 μL undiluted specimen and 5–10 μL of samples serially diluted in sterile distilled water were dropped onto agar plates. Limit of bacterial detection was 100 CFU/mL. Mice were monitored twice daily after infection for clinical signs of severe infection/sepsis including ruffled fur, hunched posture, respiratory distress and reduced activity. Mice were humanely euthanized either when the aforementioned signs of severe sepsis were observed or at the termination of the experiment by carbon dioxide asphyxiation followed by cervical dislocation. No unexpected deaths occurred.

### Statistical analyses

For OPA, OPK, CDA and determining bacterial organ load *in vivo*, unpaired Student’s t-test was applied to compare the activity of control and anti-PspA mAbs. To compare survival curves, Mantel-Cox tests were applied. All statistical analyses were performed using GraphPad Prism (GraphPad Software)

## Results

### Generation of broadly reactive anti-PspA mAbs

Anti-PspA mAbs were generated by immunization of BALB/c mice (n = 48) with recombinant PspA derived from *S*. *pneumoniae* D39 (PspA family 1, clade 2) or TIGR4 (Family 2, clade 3). Some mice received booster injections with recombinant PspA from other *S*. *pneumoniae* strains. The PspA proteins contained the α-helical and proline-rich region (PRR), but not the choline-binding domain.

Splenocytes from immunized mice were fused with mouse myeloma cells to generate hybridomas and single hybridoma clones secreting anti-PspA mAbs were isolated. After passing primary screening (binding to protein antigens and several *S*. *pneumoniae* strains), mAbs were then tested for binding to 28 *S*. *pneumoniae* strains covering PspA clades 1–5. 11 mouse anti-PspA IgG mAbs displaying binding to 68–96% of the strains were obtained (Table 2). All mAbs derived from mice immunized with Family 1 PspA cross-reacted with strains expressing Family 2 PspA and vice versa. Moreover, all mAbs bound to at least one of three PspA deficient strains indicating that immunization with recombinant PspA induced mAbs that cross-reacted with non-PspA proteins (Table 2).

**Table 2:** Binding spectrum of mouse anti-PspA mAbs.

	*S*. *pneumoniae* strains tested				Binding of anti-PspA mAb candidates to 28 *S*. *pneumoniae* wild-type and 3 PspA-deficient mutant strains										
#	Strain	Serotype	PspA Family	PspA Clade	139F3	139G3	139I3	139I6	139I8	140G1	140G5	140G6	140G11	140H1	140H4
1	ATCC-6301	1	1	1	**Y**	**Y**	**Y**	**Y**	**Y**	**Y**	**Y**	**Y**	**Y**	**Y**	**Y**
2	NCTC-11910	23F	1	1	**-**	**-**	**-**	**-**	**-**	**-**	**-**	**-**	**-**	**Y**	**-**
3	ATCC-49136	17	1	1	**-**	**Y**	**Y**	**Y**	**Y**	**Y**	**Y**	**Y**	**Y**	**Y**	**Y**
4	ATCC-49619	19F	1	1	**Y**	**Y**	**Y**	**Y**	**Y**	**Y**	**Y**	**Y**	**Y**	**Y**	**Y**
5	NCTC-11888	6B	1	1	**Y**	**Y**	**Y**	**Y**	**Y**	**Y**	**Y**	**Y**	**Y**	**Y**	**Y**
6	EF3030	19	1	1	**-**	**-**	**Y**	**Y**	**Y**	**Y**	**Y**	**Y**	**Y**	**Y**	**Y**
7	BAA-658	6B	1	1	**Y**	**Y**	**Y**	**Y**	**Y**	**Y**	**Y**	**Y**	**Y**	**Y**	**Y**
8	ATCC-700675	6B	1	1	**Y**	**Y**	**Y**	**Y**	**Y**	**Y**	**Y**	**Y**	**Y**	**Y**	**Y**
9	ATCC-6305	5	1	2	**-**	**-**	**Y**	**Y**	**Y**	**Y**	**Y**	**Y**	**Y**	**Y**	**Y**
10	WU2	3	1	2	**-**	**-**	**Y**	**Y**	**Y**	**Y**	**Y**	**Y**	**Y**	**Y**	**Y**
11	NCTC-7978	3	1	2	**-**	**-**	**Y**	**Y**	**Y**	**Y**	**Y**	**Y**	**Y**	**Y**	**Y**
12	NCTC-7466 (D39)	2	1	2	**-**	**-**	**Y**	**Y**	**Y**	**Y**	**Y**	**Y**	**Y**	**Y**	**Y**
13	NCTC-11886	4	2	3	**Y**	**Y**	**Y**	**Y**	**Y**	**Y**	**Y**	**Y**	**Y**	**Y**	**Y**
14	BAA-475	19A	2	3	**-**	**Y**	**Y**	**Y**	**Y**	**Y**	**Y**	**Y**	**Y**	**Y**	**Y**
15	BAA-340	14	2	3	**Y**	**Y**	**Y**	**-**	**Y**	**Y**	**-**	**-**	**Y**	**Y**	**Y**
16	ATCC-700905	19F	2	3	**Y**	**Y**	**Y**	**Y**	**Y**	**Y**	**Y**	**Y**	**Y**	**Y**	**Y**
17	PJ-1324	6B	2	3	**Y**	**Y**	**Y**	**Y**	**Y**	**Y**	**Y**	**Y**	**Y**	**Y**	**Y**
18	TIGR4	4	2	3	**Y**	**Y**	**Y**	**Y**	**Y**	**Y**	**Y**	**Y**	**Y**	**Y**	**Y**
19	NCTC-11902	14	2	4	**Y**	**Y**	**Y**	**Y**	**Y**	**Y**	**Y**	**Y**	**Y**	**Y**	**Y**
20	NCTC-11905	18C	2	4	**Y**	**Y**	**Y**	**Y**	**Y**	**Y**	**Y**	**Y**	**Y**	**Y**	**Y**
21	NCTC-11906	19F	2	4	**Y**	**Y**	**Y**	**Y**	**Y**	**Y**	**Y**	**Y**	**Y**	**Y**	**Y**
22	ATCC-700673	19A	2	5	**Y**	**Y**	**Y**	**Y**	**Y**	**Y**	**Y**	**Y**	**Y**	**Y**	**Y**
23	ATCC-6303	3	2	5	**Y**	**Y**	**Y**	**Y**	**Y**	**Y**	**Y**	**Y**	**Y**	**-**	**Y**
24	NCTC-11897	9V	2	5	**Y**	**Y**	**Y**	**Y**	**Y**	**Y**	**Y**	**Y**	**Y**	**Y**	**Y**
25	BAA-612	6B	2	5	**Y**	**Y**	**Y**	**Y**	**Y**	**Y**	**Y**	**Y**	**Y**	**Y**	**Y**
26	ATCC-49150	?	?	?	**-**	**Y**	**Y**	**Y**	**Y**	**Y**	**Y**	**Y**	**Y**	**Y**	**Y**
27	ATCC-700671	9V	?	?	**Y**	**Y**	**Y**	**Y**	**Y**	**Y**	**Y**	**Y**	**Y**	**Y**	**Y**
28	DS2341-94	4	?	?	**Y**	**Y**	**-**	**-**	**-**	**-**	**-**	**-**	**-**	**Y**	**Y**
	D39 PspA mutant	2	1	2	**-**	**-**	**Y**	**Y**	**Y**	**Y**	**Y**	**Y**	**Y**	**Y**	**Y**
	PJ-1324 PspA mutant	6B	2	3	**Y**	**Y**	**Y**	**Y**	**Y**	**Y**	**Y**	**Y**	**Y**	**Y**	**Y**
	TIGR4 PspA mutant	4	2	3	**-**	**-**	**Y**	**Y**	**Y**	**Y**	**Y**	**Y**	**Y**	**Y**	**Y**
		# of wild-type strains bound			19/28	21/28	26/28	25/28	26/28	26/28	25/28	25/28	26/28	27/28	27/28
		Overall binding (%)			68%	75%	93%	89%	93%	93%	89%	89%	93%	96%	96%

Binding of 11 mouse anti-PspA mAbs to 28 *S*. *pneumoniae* wild-type strains and PspA-deficient mutants was determined. Live pneumococci were incubated with anti-PspA mAbs or isotype negative control antibodies (= primary antibodies) or antibody vehicle only. Primary antibody binding was detected with PE-labeled secondary antibody. The fluorescence intensity of the bacterial particles was measured by flow cytometry. Y in cell: Anti-PspA mAb bound;—in cell: Anti-PspA mAb did not bind relative to vehicle and isotype control negative control samples.

Next, the reactivity of the 11 anti-PspA mAbs to 20 *S*. *pneumoniae* strains, which were previously used to investigate the genetic basis for serologic PspA diversity [8] was evaluated. The mAbs bound to 50–100% of the strains with 140H1 having the broadest reactivity (S1 Table).

From these activities, four lead mAbs were identified: 139G3 (IgG2a) bound to 32 of the 48 strains tested (Coverage: 67%), 140G1 (IgG1) and 140G11 (IgG2a) had an identical binding pattern and reacted with 43/48 strains (90% coverage), 140H1 (IgG1) bound to 47/48 strains (98% coverage).

### Epitope mapping for lead anti-PspA mAbs

**Fig 1:**
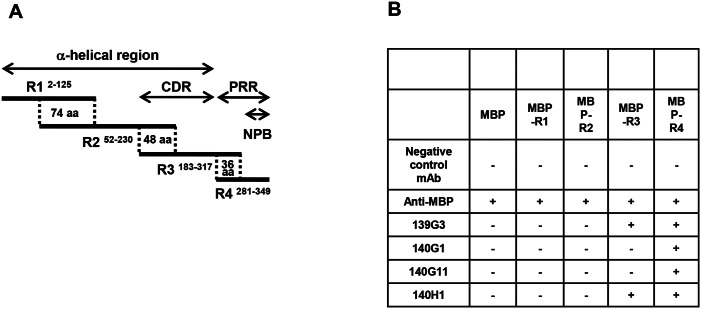
Epitope regions of lead anti-PspA mAbs. To determine the epitope regions of lead mAbs, PspA fragments linked to maltose-binding protein (MBP-R1-R4) with overlapping regions were prepared. (A) The locations of MBP-R1-R4 in BAA-658 PspA are schematically depicted; superscripted, the amino acids (aa) of PspA encompassed by the fragments are indicated. Dotted lines mark overlapping fragment regions. CDR, Clade Defining Region; PRR, proline-rich region; NPB, non-proline block. (B) Binding of anti-PspA mAbs to MBP (negative control) and MBP-R1 to MBP-R4 was tested by ELISA. An anti-MBP antibody served as positive control. The table summarizes the data. +, mAb binding observed; -, binding not observed.

To further characterize the lead mAbs, their epitope region was determined by testing their binding to four overlapping PspA fragments conjugated to maltose-binding protein (MBP-R1 to MBP-R4) of BAA-658 PspA (Family 1, clade 1) spanning the N-terminal α-helical coiled-coil and PRR regions including the non-proline block (NPB) (Fig 1A).

As summarized in Fig 1B, 140G1 and 140G11 only reacted with MBP-R4 indicating that their epitopes were located in the NPB. Consistent with this, these mAbs did not bind to several *S*. *pneumoniae* strains lacking an NPB [8] (S1 Table). 139G3 and 140H1 reacted with MBP-R3 and -R4 suggesting specificity for the overlapping part of these fragments. ELISAs with 15–19 amino acid peptides mapped the epitopes of 139G3 and 140H1 to the PRR sequences TPAPAPKPEQPA and KPAPAPQP, respectively.

### Binding comparison of the lead anti-PspA mAbs

In order to further define the breadth and strength of activity for the four lead anti-PspA mAbs, binding strength to PspA clade 1–3 proteins derived from *S*. *pneumoniae* BAA-658, D39, and TIGR4 were compared by BIAcore (surface plasmon resonance (SPR)) and analyzed using a bivalent binding model. Only 140H1, showed strong binding to all PspA proteins with KD values as low as 0.8 nM (S2 Table).

### Anti-PspA mAbs conferred C3 deposition and showed strong activity in opsonophagocytic killing (OPK) assays with human phagocytes

Complement is known to be critical for the human host defense during invasive pneumococcal disease [18]. The classical pathway of complement activation is initiated by antibody-mediated antigen recognition leading to the deposition of C3b-iC3b on *S*. *pneumoniae* resulting in complement receptor mediated uptake and destruction of the bacteria by phagocytes [19]. Complement deposition assays (CDAs) and OPK experiments with anti-pneumococcal antibodies and hyper-immune sera have been proposed as surrogates of protection assays that can predict the protective efficacy of anti-*S*. *pneumoniae* antibodies and vaccines, respectively [20–22].

Mouse IgG2a, the counterpart of human IgG1, is more potent than mouse IgG1 in binding C1q and thus activating the classical complement pathway [23]. To facilitate an appropriate comparison of the *in vitro* and *in vivo* activities of the anti-PspA mAbs, mouse IgG2a (class-switched) versions of 140G1 (140csG1) and 140H1 (140csH1) were generated for mAb activity testing.

**Fig 2:**
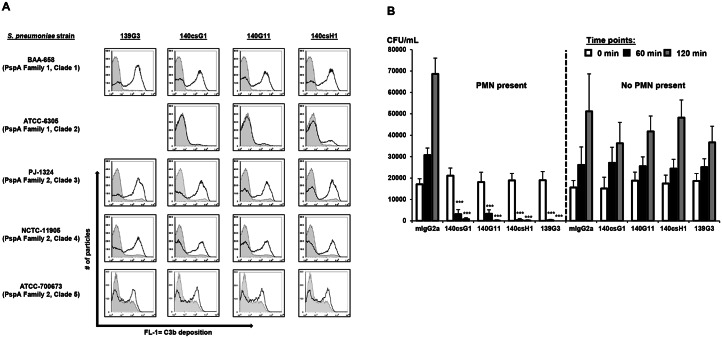
Anti-PspA mAbs show activity in complement deposition and opsonophagocytic killing assays. (A) Pneumococci were incubated with 0.25–5 μg/mL mouse isotype control or anti-PspA IgG2a mAbs and mouse serum. Bound C3 was detected with a FITC-labeled anti-mouse C3 antibody using flow cytometry. Histograms: FL-1 for isotype (tinted; gray lines) or the respective anti-PspA (black lines) mAbs. Note: 139G3 did not bind to ATCC-6305. (B) *S*. *pneumoniae* PJ-1324 cells were pre-opsonized with 1 μg/mL mouse IgG2a control or anti-PspA mAbs in 10% baby rabbit complement and 10^6^ PMN-like HL-60 cells or vehicle. At the indicated times, CFU were enumerated. Samples were run in triplicate or quadruplicate and mean values + SD of one representative experiment of several performed are shown (***, *p*<0.0005; anti-PspA vs. isotype mAb, unpaired t-test).

CDAs with mouse serum and nine *S*. *pneumoniae* strains representing PspA clades 1–5 were conducted to determine if the four mouse anti-PspA mAbs confer C3b deposition on live pneumococci. MAbs mediated C3b deposition in a strain specific manner (S3 Table). Notably, only 140csH1 was able to mediate C3b deposition on all tested bacterial strains, demonstrating high activity with ATCC-49619 (clade 1), BAA-658 (clade 1), D39 (clade 2), PJ-1324 (clade 3), NCTC-11905 (clade 4) and moderate activity with ATCC-700673 (clade 5) and ATCC-6305 (clade 2). Typical CDA results for one strain each for PspA clades 1–5 are shown in Fig 2A.

To test whether this complement opsonization translated to increased bacterial cell killing, OPK assays were performed with differentiated, PMN-like HL-60 cells [17]. In these assays, all four lead anti-PspA mAbs mediated a >2-log colony forming unit (CFU) reduction of PJ-1324 that was dependent on effector cells (Fig 2B) and complement (data not shown). While anti-PspA mAbs could not confer PJ-1324 uptake by human PMN in the absence of complement, this complement-dependence was found to be strain specific, e.g. anti-PspA mAb opsonized D39 could be readily phagocytosed without complement (data not shown).

### 139G3 and 140csH1 displayed the broadest *in vivo* efficacy

**Fig 3:**
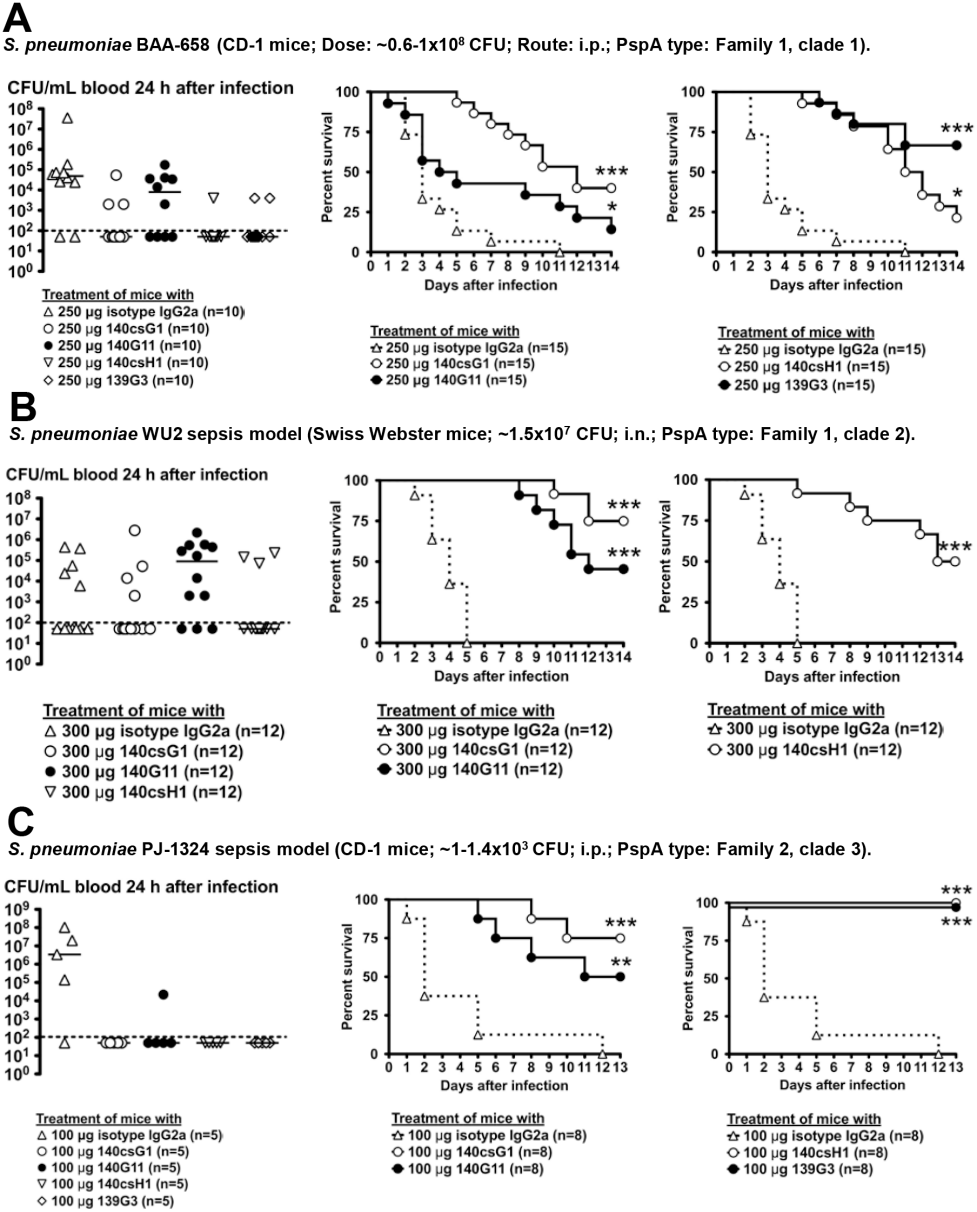
Activity of lead anti-PspA mAbs in mouse sepsis models with *S*. *pneumoniae* strains BAA-658, WU2 and PJ-1324. 4–6 h before i.p. or i.n. infection with *S*. *pneumoniae*, CD-1 or Swiss-Webster mice were pretreated i.p. with the indicated mAbs. In most experiments, heparinized tail vein blood was collected and bacterial CFU enumerated (Left panels). The results of one to two independent experiments are shown (dotted line = 100 CFU/mL, limit of detection) (*, *p*<0.05; CFU isotype vs. anti-PspA mAb treatment, unpaired t-test). Survival was followed for 13–14 days. Combined survival results of two to three independent experiments are shown (*, *p*<0.05; **, *p*<0.005; ***, *p*<0.0005, isotype vs. anti-PspA mAbs, Mantel-Cox test).

**Fig 4:**
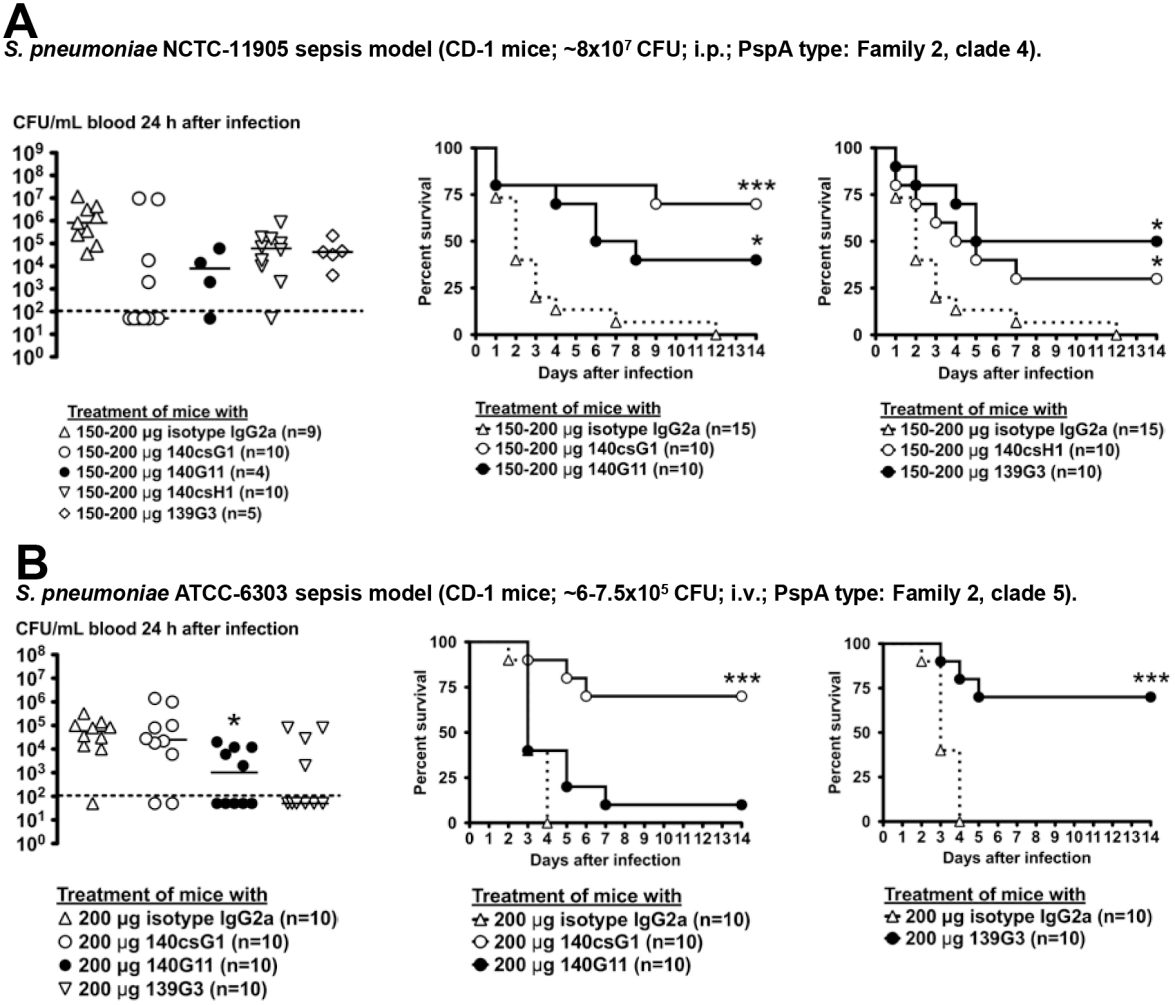
Activity of lead anti-PspA mAbs in mouse sepsis models with *S*. *pneumoniae* strains NCTC-11905 and ATCC-6303. 4–6 h before i.p. or i.v. infection with *S*. *pneumoniae*, CD-1 mice were pretreated i.p. with the indicated mAbs. In most experiments, heparinized tail vein blood was collected and bacterial CFU enumerated (Left panels). The results of one to two independent experiments are shown (dotted line = 100 CFU/mL, limit of detection) (*, *p*<0.05; CFU isotype vs. anti-PspA mAb treatment, unpaired t-test). Survival was followed for 13–14 days. Combined survival results of two to three independent experiments are shown (*, *p*<0.05; **, *p*<0.005; ***, *p*<0.0005, isotype vs. anti-PspA mAbs, Mantel-Cox test).

Next, *in vivo* efficacy of the lead candidates was tested in up to 13 mouse passive immunization models in which the mAbs were applied shortly before infection. Results with *S*. *pneumoniae* strains representing PspA clades 1–5 are shown in Figs 3 and 4.

All anti-PspA mAbs displayed statistically significant efficacy in the BAA-658 intraperitoneal (i.p.) infection model with 139G3 having the strongest activity (Fig 3A). In most experiments, blood bacterial burden was determined 24 h after infection; mice treated with anti-PspA mAbs had lower median blood CFU versus isotype-treated animals, but statistical significance was not reached.

140csG1, 140G11 and 140csH1 protected mice from mortality in an intranasal challenge model with highly encapsulated WU2 [24]; 24 h after infection, no statistically significant differences in blood CFU were observed (Fig 3B). 139G3 was not tested as it does not bind WU2.

All mAbs had activity in a PJ-1324 i.p. infection model with 139G3 and 140csH1 conferring 100% survival; 24 h after infection, no bacteria were detected in the blood of most of the anti-PspA treated mice, whereas 80% of isotype-treated animals had between 10^5^−10^8^ CFU/mL blood (Fig 3C).

Passive immunization with all mAbs significantly reduced mortality in animal experiments with NCTC-11905 and median blood CFU in anti-PspA treated mice was lower than in control mice, though statistical significance was not reached (Fig 4A).

Although 140G11 lacked efficacy in ATCC-6303 experiments, 139G3 and 140csG1 conferred strong protection; interestingly, 140G11 treated mice had significantly lower blood CFU counts as compared to control mice which, in this case, did not correlate with a survival advantage (Fig 4B). 140csH1 activity was not tested as it does not bind to ATCC-6303.

The results of all mouse passive immunization experiments are summarized in S4 Table. 139G3 showed activity in 8/9 animal models (89%), 140csG1 in 8/13 (62%), 140G11 in 7/13 (54%), and 140csH1 in 9/11 (82%).

Based on all collected *in vitro* and *in vivo* data, 140csH1 was prioritized for further characterization.

### Combination treatment has the potential to increase efficacy of anti-PspA mAbs and standard-of-care antibiotics

**Fig 5:**
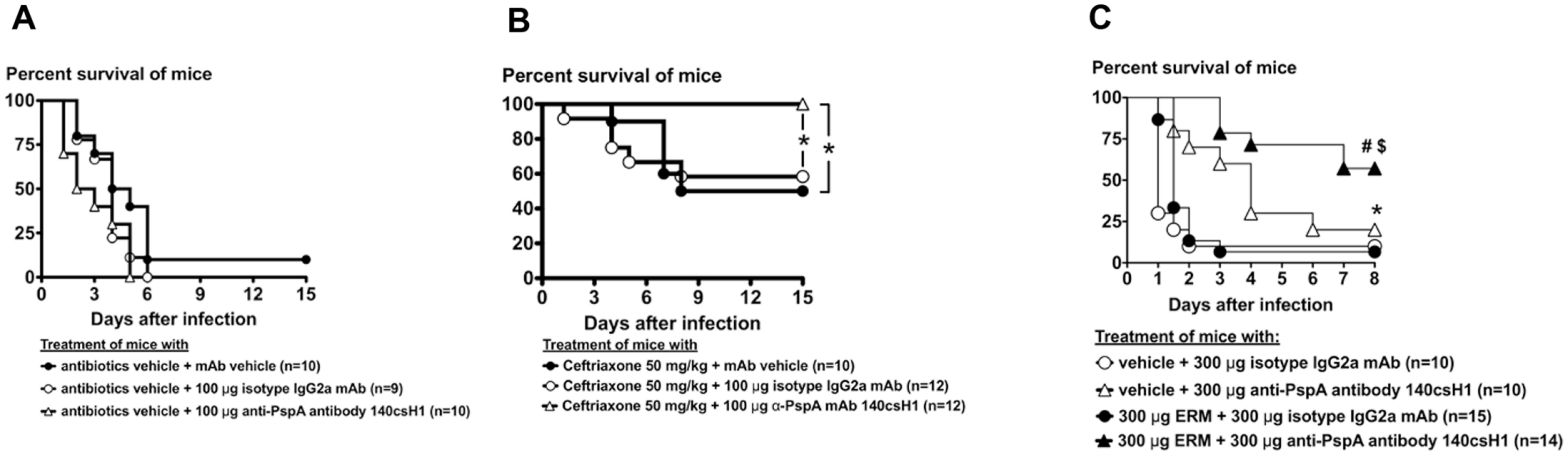
Combination treatment can potentially increase efficacy of anti-PspA mAbs and standard-of-care antibiotics. (A-B) 24 h after i.p. infection with ~2.1–3.2x10^4^ CFU of exponential phase PJ-1324 (~21- to 32-fold LD_100_), CD-1 mice were treated i.p. with 200 μL PBS alone (vehicle), or 200 μL PBS containing 100 μg isotype IgG2a mAb or 140csH1 ± 1 mg ceftriaxone (~50 mg/kg). Survival was followed for 15 days after infection. Combined results of three independent experiments are shown. *, *p*<0.05, Mantel-Cox test. (C) 30 min after i.p. infection with 3.2–6.5x10^7^ CFU exponential phase BAA-658 (ERM resistant), groups of 4–5 female CD-1 mice were treated i.p. with 200 μl of PBS containing 300 μg of mouse isotype IgG2a antibody or 140csH1. Mice were immediately treated intragastrically with 200 μL PBS, 1.5% ethanol (vehicle) or 200 μL PBS, 1.5% ethanol, 1.5 mg/mL ERM (Dose: 15 mg/kg). Survival was monitored for 8 days. Combined results of three independent experiments are shown. *, *p*<0.05 vehicle + isotype vs. vehicle +140csH1; ^#^, *p*<0.04 vehicle + 140csH1 vs. ERM + 140csH1; ^$^, *p*<0.0001 ERM + isotype vs. ERM + 140csH1, Mantel-Cox test.

We hypothesized that 140csH1 could exert additive activity when applied in conjunction with standard-of-care antibiotics used for treating pneumococcal infections. To test this, 140csH1 was administered in combination with the 3^rd^ generation cephalosporin ceftriaxone in a systemic infection model in which mice were infected with a >20-fold absolute lethal dose (LD_100_) of *S*. *pneumoniae* PJ-1324. Under conditions in which a single dose of 100 μg 140csH1 administered 24 hours after infection lacked activity (Fig 5A) and a 50 mg/kg dose of ceftriaxone conferred around 50% survival (Fig 5B), a combination of ceftriaxone and 140csH1 showed strong efficacy and rescued all mice indicating a therapeutic benefit of combination treatment.

In addition to providing a potential synergistic benefit when used in combination with standard of care therapy, it was important to test 140csH1 for efficacy against an antibiotic resistant strain of *S*. *penumoniae*, given that the emergence of pneumococci resistant to antibiotics, e.g. macrolides, which are commonly given to treat pneumonia, is of concern [25, 26]. To this end, we tested whether 140csH1 confers protection in a mouse sepsis model using multi-drug resistant *S*. *pneumoniae* BAA-658 in which the macrolide erythromycin (ERM) was expected to fail.

A combination of 15 mg/kg ERM and 300 μg isotype mAb did not have significant activity compared to vehicle/isotype treatment as BAA-658 is high-level ERM-resistant [27] (Fig 5C). In contrast, a therapeutically administered combination of vehicle and 140csH1 conferred significant protection versus vehicle/isotype treatment. Unexpectedly, mouse survival was further improved when 140csH1/ERM combinations were given, which may be due to minor activity of ERM against BAA-658 and/or anti-inflammatory effects macrolides are known to have [28].

### Therapeutic efficacy of anti-PspA mAb 140csH1 in a mouse lung infection model

**Fig 6:**
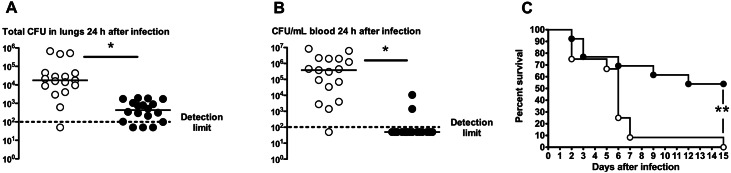
Therapeutically administered 140csH1 improves outcome in mouse lung infection model with PJ-1324. (A-B) 6 h or (C) 24 h after i.t. infection with 0.8–2.7x10^6^ CFU of PJ-1324 in 50 μL PBS, CD-1 mice were treated i.p. with 200 μg isotype IgG2a mAb (open circles) or 140csH1 (closed circles). 24 h after infection, (A.) lung and (B.) tail vein blood CFU were determined (dotted line = detection limit of 100 CFU/mL). Combined results of three independent experiments are shown. *, *p*<0.04, isotype vs. 140csH1 treated mice, unpaired t-test. (C) Mice were treated 24 h after infection with isotype (open circles; n = 12 total) or 140csH1 (closed circles; n = 13 total). Survival was followed for 15 days after infection. Combined results of two independent experiments are shown. **, *p*<0.004, isotype vs. 140csH1 survival curve, Mantel-Cox test.

Next, the therapeutic activity of 140csH1 was tested in a pneumonia model where mice were infected intratracheally with *S*. *pneumoniae* PJ-1324 followed 6 hours later by administration of 140csH1. Such therapeutic treatment resulted in >97% reduced median lung CFU (~5.2x10^2^ CFU/lung) as compared to isotype control antibody treated mice (~1.8x10^4^ CFU/lung) (Fig 6A). A median of 3.8x10^5^ CFU/mL blood were detected in isotype treated mice 24 hours after the infection, while most mice receiving 140csH1 had no detectable bacteria in their blood (Fig 6B) indicating that 140csH1 inhibited the spread of PJ-1324 to extra-pulmonary sites in the majority of mice 24 hours post-infection. Finally, 140csH1 significantly protected mice from mortality (Fig 6C) when administered 24 h after infection in the pneumonia model: 7/13 (~54%) of 140csH1 treated mice survived while all isotype treated animals succumbed to infection, further substantiating the therapeutic potential of anti-PspA mAbs.

### Preparation of chimeric 140H1 and enhancement of anti-PspA effector function

We were interested in whether or not modifications to the Fc domain of our anti-PspA antibody would be able to confer additional effector function to the mAb, specifically, enhanced C3b deposition and increased affinity of the Fc portion of the antibody to Fc gamma receptor IIIb (FcγRIIIb). With respect to enhanced C3b deposition, previous reports have demonstrated that effector function of anti-cancer antibodies can be improved by Fc domain alterations. A human IgG1/IgG3 anti-CD20 antibody showed higher cytotoxicity (versus hIgG1) towards cancer cells through increased classical complement pathway activation [15]. In regards to enhancing mAb Fc affinity to FcγRIIIb, it is known that the Asn-linked sugar chain which binds on Fc domain of human IgG1 is normally fucosylated. Non-fucosylated anti-lymphoma hIgG1 has been shown to have higher affinity to FcγRIIIb than fucosylated comparator antibodies thereby potentiating cancer cell phagocytosis by human PMN [29]. We therefore hypothesized that hIgG1/IgG3 anti-PspA mAbs may confer enhanced C3b deposition on the surface of pneumococci over IgG1 antibodies and that non-fucosylated anti-PspA mAbs may confer increased uptake of pneumococci by human phagocytes.

To address these hypotheses, four chimeric 140H1 versions were prepared containing the mouse 140H1 variable region and human IgG (hIgG) constant region as follows: (1) fucosylated hIgG1 (Fu+ hIgG1); (2) non-fucosylated hIgG1 (Fu- hIgG1); (3) fucosylated hIgG1/IgG3 (Fu+ hIgG1/IgG3); or (4) non-fucosylated hIgG1/IgG3 (Fu- hIgG1/IgG3).

**Fig 7:**
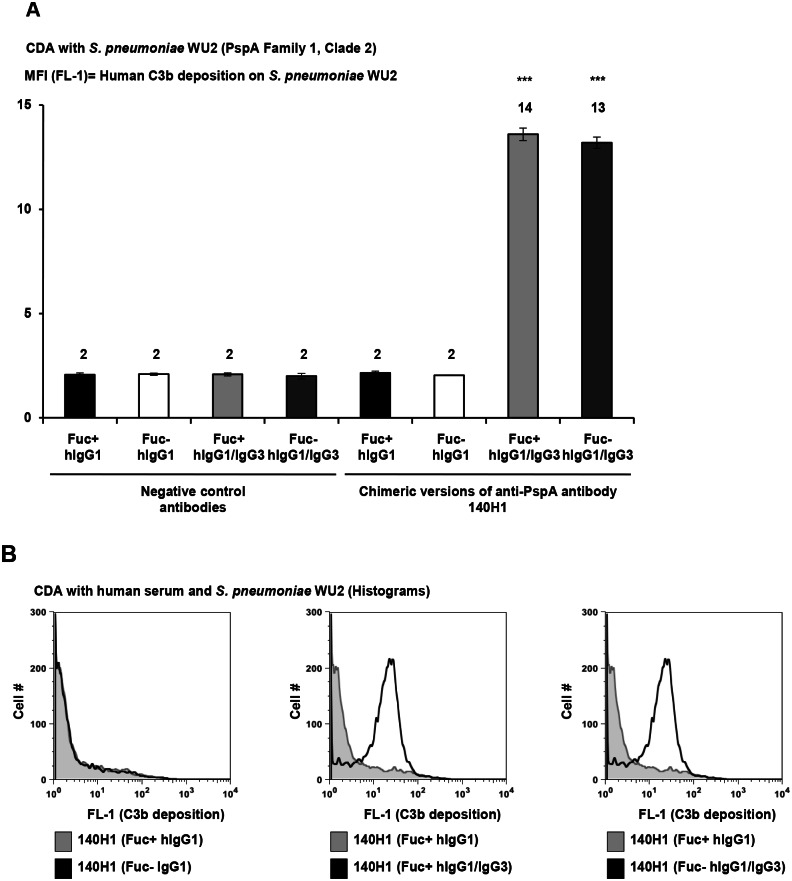
Increased activity of human IgG1/IgG3 versions of chimeric anti-PspA antibodies in CDAs with human serum (WU2). (A-B) 10^8^ CFU/mL of highly encapsulated WU2 were incubated for 15 min in 2.5% unabsorbed human serum pool ± 5 μg/mL of control or chimeric anti-PspA antibodies. Subsequently, C3b deposition was detected with FITC-labeled anti-C3 antibody. Samples were run in triplicate and the FL-1 intensity of the cells measured by flow cytometry. (A) Average MFI values ± SD of one representative experiment of two performed with similar results are shown. ***, *p*<0.0005, vs. chimeric Fu+ and Fu- hIgG1 versions of 140H1, unpaired Student’s t-test. (B) FL-1 data for 10,000 bacterial particles

**Fig 8:**
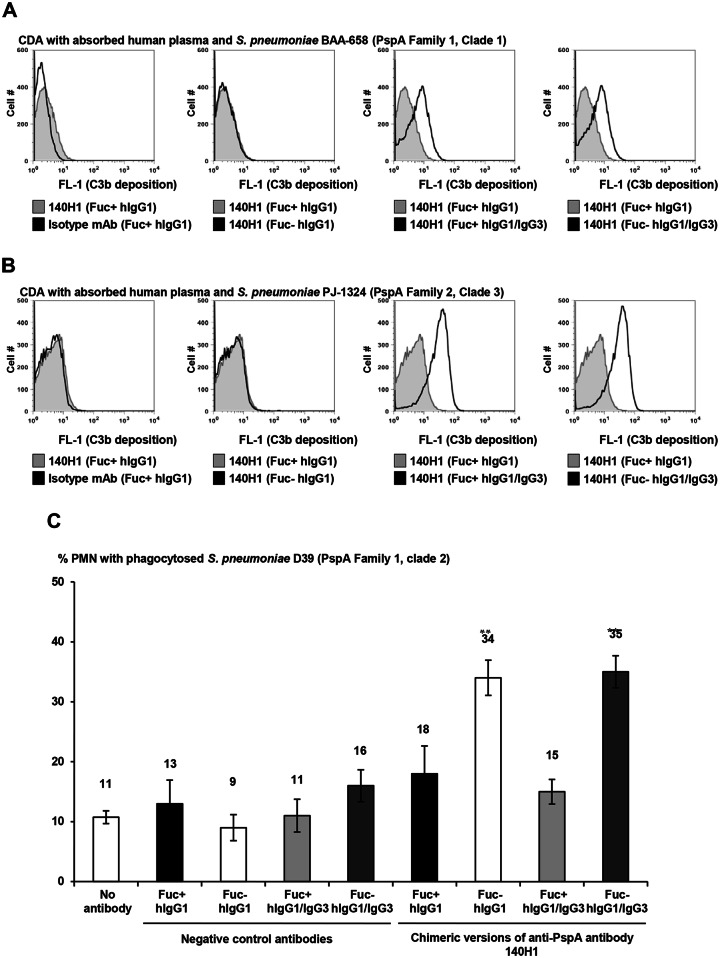
Increased activity of human IgG1/IgG3 versions of chimeric anti-PspA antibodies in CDAs with human serum (BAA-658 and PJ-1324) and of non-fucosylated chimeric anti-PspA antibodies in OPAs with human phagocytes. (A-B) 10^8^ CFU/mL of BAA-658 and PJ-1324 were incubated for 15 min in 40% absorbed human plasma ± 5 μg/mL of control or chimeric anti-PspA antibodies. Subsequently, C3b deposition was detected with FITC-labeled anti-C3 antibody. Samples were run in triplicate and the FL-1 intensity of the cells measured by flow cytometry, using 20,000 analyzed particles per sample. (A) Average MFI values ± SD of one representative experiment of two performed with similar results are shown. ***, *p*<0.0005, vs. chimeric Fu+ and Fu- hIgG1 versions of 140H1, unpaired Student’s t-test. (C) OPAs. FITC-labeled D39 particles were incubated for 30 min (5:1) with human blood PMN ± 10 μg/mL isotype control or chimeric anti-PspA antibodies. Subsequently, 200 PMN/sample were analyzed by fluorescence microscopy for phagocytosis of pneumococci. Ethidium bromide was used to differentiate between intra- and extracellular pneumococci. Samples were run in quadruplicate and average values ± SD of one representative experiment of two performed with similar results are shown (**, *p*<0.005, vs. chimeric IgG1 version of 140H1, unpaired Student’s t-test).

140H1 chimeric antibodies were compared in C3 deposition assays with three *S*. *pneumoniae* strains and human serum or plasma as complement source. Both Fu+ and Fu- hIgG1/IgG3 140H1 conferred ~6-fold increased C3 deposition on highly encapsulated WU2 as compared to Fu+ and Fu- comparator hIgG1 (Fig 7A and 7B). This showed that hIgG1/IgG3 type anti-PspA antibodies conferred greater C3 deposition than IgG1 irrespective of their fucosylation status. Further validating this, the Fu+ and Fu- hIgG1/IgG3 type 140H1 had higher activity in CDAs with PJ-1324 and BAA-658 (Fig 8A and 8B). Of note, the assays with the latter two strains were conducted with absorbed human plasma because of high background activity of the human serum pool in CDAs with these strains.

In phagocytosis assays without complement, Fu- hIgG1 and hIgG1/IgG3 140H1 versions conferred ~2-fold better uptake of D39 cells by human PMN versus the respective Fu+ IgG1 and IgG1/IgG3 molecules, showing that the degree of anti-PspA antibody activity in complement-independent OPAs was heavily dependent on their fucosylation status (Fig 8C). Of note, chimeric anti-PspA antibodies could not mediate significant uptake of PJ-1324 or BAA-658 cells by human PMN in the absence of complement (data not shown) suggesting that an interaction between antibody bound to the pneumococcal surface and Fc receptors on PMN was impaired in these strains.

Taken together, the results obtained with chimeric 140H1 demonstrated that the *in vitro* activity of anti-PspA antibodies in CDAs and OPAs can be significantly enhanced through antibody engineering and suggest that Fu- hIgG1/IgG3 anti-PspA mAbs could exhibit higher therapeutic efficacy compared to Fu+ hIgG1-type antibodies. Similar CDA and OPA results were obtained with chimeric 140G1 (S1 Fig).

Therapeutic use of mouse/human chimeric antibodies may be limited because of the potential to induce a human anti-mouse antibody response (HAMA) [30]. To further explore the potential of development of 140H1 for therapeutic use, the antibody was humanized. Humanized variants were tested for binding to PspA by BIAcore, binding to live *S*. *pneumoniae* cell surface and OPA activity with human PMNs. Several humanized versions of 140H1 retained functionality as compared to the original mouse antibody (data not shown) demonstrating the potential for future therapeutic development of such antibodies.

## Discussion

Several studies indicated PspA as a promising vaccine candidate [2, 31]. Previous evidence for the potential of PspA as a target for passive immunotherapy came from mouse studies showing protective efficacy of PspA specific hyper-immune sera and mAbs generated in mice or human [7, 10, 32–35]. However, these studies did not look at broad-spectrum strain coverage and broad *in vitro* and *in vivo* activity of anti-PspA mAbs.

This paper described the generation of the broadly reactive anti-PspA mouse mAb 140H1 which bound to 98% of 48 *S*. *pneumoniae* strains tested, including representatives of the clinically most relevant PspA clades 1–5, serotypes most associated with invasive disease, and multi-drug resistant strains.

The broad binding spectrum of 140H1 can be explained by the fact that the epitope recognized is located in the PRR of PspA which is well conserved across the PspA families [8]. It has been shown that the degree of cross-reactivity of serum from mice immunized with recombinant PspA molecules was dependent on both the α-helical and PRR regions [31] and that PRR antigen in vaccines has the potential to provide cross-protection against a broad spectrum of pneumococcal strains [36].

The binding of 140H1 to PspA mutant strains was likely due to antigenic cross-reactivity of the PRRs of PspA and PspC [10, 37] or other proteins. A recent study of antibodies elicited in children colonized with *S*. *pneumoniae* demonstrated that antibodies are produced that are able to recognize PRR region of PspA cloned from AC094 (Family 1, Clade 1) irrespective of the PspA family of the infecting strain [36] further indicating a potential for broad cross-reactivity of antibodies. Moreover, our results support previous studies that suggest potential of the PspA PRR for an active immunization approach [10, 31]. The highly conserved nature of PspA PRR and the cross-reactivity of 140H1 with non-PspA antigens on the pneumococcal surface may reduce the possibility that evasion mutants would arise from use of this mAb in patients.

In regards to the novelty of the 140H1 epitope, the Briles laboratory published anti-PspA mAbs originating from mice immunized with recombinant PspA molecules [10, 33]. Three of the mAbs, PR-1A4.7, PR-6A5.12, and K67, were shown to be specific for the NPB, whereas the epitope of a fourth described mAb, PR-5C4.7, was located C-terminally of the NPB. The 140H1 epitope was found to be N-terminal of the NPB and to our knowledge is novel.

Since 140H1 bound to almost all *S*. *pneumoniae* strains tested, the epitope(s) it reacts with seem to be more conserved than the NPB. It was previously reported that only 14/24 tested PspA sequences contained the NPB [8] providing additional evidence that antibodies to this region may not be as broadly reactive as 140H1.

140csH1 displayed activity in assays considered to be surrogates of protection. 140csH1 had the broadest activity of the four lead anti-PspA mAbs in C3 deposition assays with *S*. *pneumoniae* strains expressing clade 1–5 PspA. In OPK experiments, 140csH1 mediated >3-log bacterial killing that was dependent on the presence of phagocytes and complement. The CDA and OPK results strongly suggested that the anti-PspA mAbs facilitated phagocytic uptake and killing through opsonizing activity by mediating complement deposition onto the bacteria. Moreover, in addition to activation of the classical complement pathway, agglutination effects by anti-pneumococcal antibodies have the potential to increase the phagocytic killing of *S*. *pneumoniae* [38].

We showed that anti-PspA antibodies with enhanced effector functions have the potential to dramatically increase opsonization, phagocytosis, and thereby killing of pneumococci which may significantly lower the therapeutic dose in patients suffering from invasive pneumococcal disease, making treatment more economically feasible. Specifically, experiments with chimeric 140H1 demonstrated that Fu- anti-PspA antibodies could mediate higher uptake of D39 in the absence of complement than Fu+ mAbs. However, chimeric 140H1 could not confer PJ-1324 or BAA-658 complement-independent phagocytosis by human PMN despite binding to these strains. We speculate that the pneumococcal capsule hindered recognition of the surface bound antibodies by Fc receptors on phagocytes. The killing of PJ-1324 by human phagocytes in the presence of anti-PspA mAbs was dependent on active complement.

These results indicate that to achieve highest efficacy, a mAb drug targeting conserved regions of PspA must be able to confer sufficient complement deposition to allow phagocytic uptake of antibody-opsonized pneumococci that are not recognized via Fc receptors. We have shown that Fu- hIgG1/IgG3 type anti-PspA antibodies effectively increased C3 deposition and thus have the greatest promise for development as therapeutics. A recent study supports this conclusion, showing that anti-PspA mAb conferring C3 deposition on pneumococci *in vitro* had a positive correlation with ability to provide passive protection in an *in vivo S*. *pneumoniae* infection model [39].

Further substantiating the potential of anti-PspA mAbs as passive immunization drugs, 140csH1 was protective in both mouse sepsis and pneumonia experiments when administered alone or in conjunction with standard-of-care antibiotics. Experiments with ceftriaxone demonstrated that anti-PspA mAbs have the potential to show therapeutic benefit when combined with a standard-of-care antibiotic under conditions in which the antibiotic alone failed to confer 100% survival. Moreover, we demonstrated that ERM and anti-PspA mAb combinations show enhanced efficacy in an *in vivo* model with ERM-resistant BAA-658. These mAb/antibiotic combination experiments indicate that anti-PspA mAbs may exert additive or synergistic efficacy in treating invasive pneumococcal disease in conjunction with antibiotics. Thus, mAb/antibiotic combinations have the potential to reduce the mortality rate, duration and cost of therapy of severe pneumococcal infections compared to standard-of-care alone.

Overall, *in vivo* experimentation provided proof-of-concept that anti-PspA mAbs with broad binding spectrum and *in vitro* activity can display wide ranging *in vivo* efficacy. Consequently, we believe that PspA has strong potential as a target for the development of a passive immunization drug for the therapy of invasive pneumococcal disease. Humanized 140H1 could be a promising candidate for further development and could be used alone or in combination with standard-of-care antibiotics.

## Supporting Information

S1 Fig Increased activity of non-fucosylated chimeric 140G1 version in OPAs with human PMN. In 96-well plates, 2.5x10^6^ FITC-labeled pneumococcal particles were incubated for 30 min at 37°C with 5x10^5^ purified human blood PMN in 200 μL HBSS/10% PBS buffer and 2.5 μg/mL isotype control or chimeric anti-PspA (140G1) versions. Ethidium bromide (0.25 mg/mL final) was added to the samples to allow differentiation between intra- and extracellular pneumococci and 200 live PMN/sample analyzed by fluorescence microscopy for bacterial uptake. Samples were run in quadruplicate and average values ± SD of one representative experiment of at least two performed are shown in the graphs. **, *p*<0.005; ***, *p*<0.0005 isotype control vs. chimeric version of 140G1, unpaired t-test.
(TIF)

S1 Table Binding of 11 mouse anti-PspA mAbs to a panel of 20 *S*. *pneumoniae* strains previously used to investigate the genetic basis for serologic PspA. Binding of 11 mouse anti-PspA mAbs to a panel of 20 *S*. *pneumoniae* wild-type strains was determined. Live pneumococci were incubated with the indicated anti-PspA mAbs or isotype negative control antibodies (primary mAbs) or antibody vehicle only. Bound primary mAbs were detected with PE-labeled secondary anti-mouse IgG antibody. Fluorescence intensity of bacterial particles was measured by flow cytometry. Y in cell: mAb binding was observed;—in cell: no mAb binding was seen relative to vehicle and isotype control negative control samples. The table also indicates whether the PspA of the tested strains contains an NPB as previously evaluated by Hollingshead *et al*. [8]. The strains used in this assay were kindly provided by Dr. Hollingshead and Dr. Briles (The University of Alabama at Birmingham).
(DOCX)

S2 Table Biacore-based evaluation of affinity of lead anti-PspA mouse antibodies to recombinant PspA-D39, PspA-BAA-658, and PspA-TIGR4. Affinity of lead anti-PspA mAbs (139G3, 140G1, 140G11, and 140H1) to recombinant PspA was measured by surface plasmon resonance (SPR) with a Biacore 3000 (GE Healthcare Bio-Sciences) using sensor chips with immobilized PspA ligand. Bivalent binding modeling was used to analyze the binding affinity of each mAb (Biacore 3000 Evaluation software, Biacore).
(DOCX)

S3 Table Anti-PspA mAbs show strain specific activity in complement deposition assays (CDAs) with pneumococcal strains representing PspA clades 1–5. The indicated pneumococcal strains were grown to exponential phase in THY, washed in HBSS, 5% BSA, then incubated in 0.2 mL HBSS, 3.75% BSA at 37°C with 600 rpm shaking with or without isotype control or anti-PspA mAbs (IgG2a) and commercially available mouse serum at concentrations that were optimized for each pneumococcal strain. After 30 min, cells were washed with ice-cold PBS, 0.5% BSA, and then resuspended in 100 μL PBS, 0.5% BSA containing 2 μg/mL fluorescein-labeled anti-mouse C3 antibody. After 30–60 min incubation at 4°C without mixing, bacteria were washed in ice-cold PBS, 0.5% BSA. Cells were fixed and subjected to flow cytometry. +++, strong C3 deposition; ++, moderate C3 deposition; +, weak C3 deposition; +/-, barely detectable activity; -, no detectable activity; n.a., not tested, since antibody does not bind to respective *S*. *pneumoniae* strain. All scoring was done using binding of negative control antibody as a comparator.
(DOCX)

S4 Table Activity of anti-PspA mAbs in mouse passive immunization models of septicemia with pneumococcal strains representing PspA clades 1–5. 4–6 h before i.p., i.n., or i.v. infection with the indicated *S*. *pneumoniae* strains, mice were pretreated i.p. with the indicated amounts of mAbs in PBS. In most experiments, heparinized tail vein blood was collected and bacterial CFU enumerated. Survival was monitored for 13–15 days. The combined survival results of 1–3 independent experiments are shown for each strain. *, *p*<0.05; **, *p*<0.005; ***, *p*<0.0005, survival curve, isotype control vs. anti-PspA antibodies with Mantel-Cox test; CFU, no significant protection in terms of survival, but significantly reduced bacterial numbers in tail vein blood observed 24 h after infection compared to negative control antibody treated mice; n.t., not tested. Please note that CD-1 mice were used for all but the WU2 passive immunization model in which Swiss Webster mice were used.
(DOCX)
