# Correction: Generation and Improvement of Effector Function of a Novel Broadly Reactive and Protective Monoclonal Antibody against Pneumococcal Surface Protein A of *Streptococcus pneumoniae*

**DOI:** 10.1371/journal.pone.0171732

**Published:** 2017-02-06

**Authors:** Sascha A. Kristian, Takayuki Ota, Sarah S. Bubeck, Rebecca Cho, Brian C. Groff, Tsuguo Kubota, Giuseppe Destito, Cécile Martin, John Laudenslager, Lilia Koriazova, Tomoyuki Tahara, Yutaka Kanda

Cécile Martin is not included in the author byline. She should be listed as the eighth author and affiliated with Kyowa Kirin Pharmaceutical Research, Inc. The contributions of this author are as follows: conceived and designed the experiments, performed the experiments, analyzed the data, contributed reagents/materials/analysis tools, wrote the paper.

Dr. Cécile Martin should also be included in the Funding section. The correct Funding information is as follows: This work was supported by Kyowa Hakko Kirin Co., Ltd. Japan (http://www.kyowakirin.com/index.html). All named authors are or were employees of Kyowa Kirin Pharmaceutical Research, Inc. or Kyowa Hakko Kirin Co., Ltd., Japan at the time of their involvement in the reported study. Kyowa Hakko Kirin Co., Ltd., Japan provided funding for the research of the study and provided support in the form of salaries for authors SK, TO, SB, RC, BG, TK, GD, CM, JL, LK, TT, and YK, but no individuals employed by the funder, other than the named authors, had any additional role in study design, data collection and analysis, decision to publish, or preparation of the manuscript. The specific roles of these authors are articulated in the ‘author contributions’ section.
