# Supplementary figures and images for: Generation and Improvement of Effector Function of a Novel Broadly Reactive and Protective Monoclonal Antibody against Pneumococcal Surface Protein A of Streptococcus pneumoniae
Source: PLoS One. 2016 May 12;11(5):e0154616. doi: 10.1371/journal.pone.0154616 (PMC4865217; doi:10.1371/journal.pone.0154616)

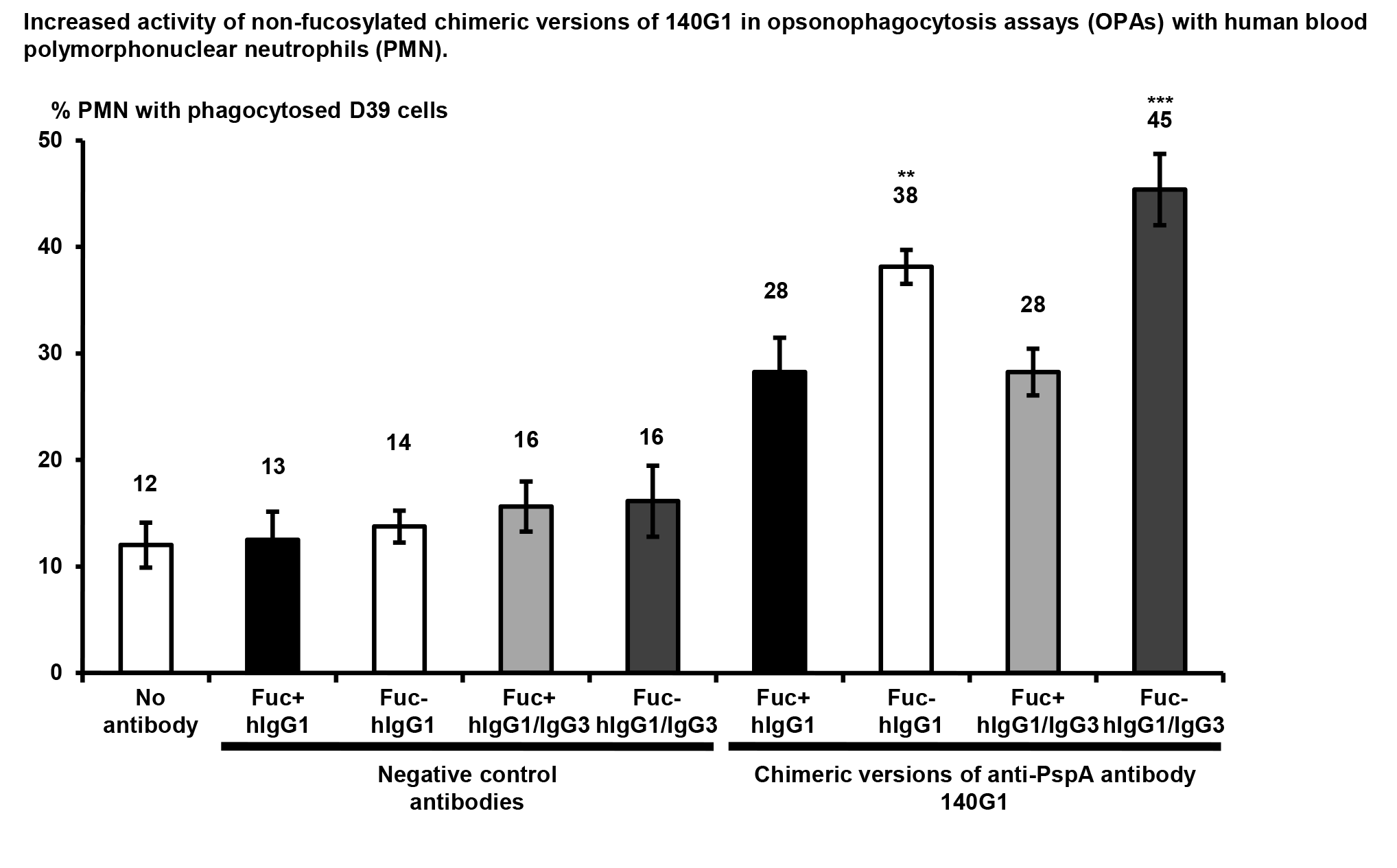

Supplement: S1 Fig — In 96-well plates, 2.5x10^6^ FITC-labeled pneumococcal particles were incubated for 30 min at 37°C with 5x10^5^ purified human blood PMN in 200 μL HBSS/10% PBS buffer and 2.5 μg/mL isotype control or chimeric anti-PspA (140G1) versions. Ethidium bromide (0.25 mg/mL final) was added to the samples to allow differentiation between intra- and extracellular pneumococci and 200 live PMN/sample analyzed by fluorescence microscopy for bacterial uptake. Samples were run in quadruplicate and average values ± SD of one representative experiment of at least two performed are shown in the graphs. **, p<0.005; ***, p<0.0005 isotype control vs. chimeric version of 140G1, unpaired t-test. (TIF) [file pone.0154616.s001.tif]
