# Supplementary material for: Generation and Improvement of Effector Function of a Novel Broadly Reactive and Protective Monoclonal Antibody against Pneumococcal Surface Protein A of Streptococcus pneumoniae
Source: PLoS One. 2016 May 12;11(5):e0154616. doi: 10.1371/journal.pone.0154616 (PMC4865217; doi:10.1371/journal.pone.0154616)
Supplement: S1 Table — Binding of 11 mouse anti-PspA mAbs to a panel of 20 S. pneumoniae wild-type strains was determined. Live pneumococci were incubated with the indicated anti-PspA mAbs or isotype negative control antibodies (primary mAbs) or antibody vehicle only. Bound primary mAbs were detected with PE-labeled secondary anti-mouse IgG antibody. Fluorescence intensity of bacterial particles was measured by flow cytometry. Y in cell: mAb binding was observed;—in cell: no mAb binding was seen relative to vehicle and isotype control negative control samples. The table also indicates whether the PspA of the tested strains contains an NPB as previously evaluated by Hollingshead et al. [8]. The strains used in this assay were kindly provided by Dr. Hollingshead and Dr. Briles (The University of Alabama at Birmingham). (DOCX) [file pone.0154616.s002.docx]

**S1 Table. Binding of 11 mouse anti-PspA mAbs to a panel of 20 *S. pneumoniae* strains previously used to investigate the genetic basis for serologic PspA diversity.**

|  | *S. pneumoniae* strains tested | | | | | Binding of mouse anti-PspA mAbs | | | | | | | | | | |
| --- | --- | --- | --- | --- | --- | --- | --- | --- | --- | --- | --- | --- | --- | --- | --- | --- |
| # | Strain | Serotype | PspA Family | PspA Clade | NPB present in PspA | 139F3 | 139G3 | 139I3 | 139I6 | 139I8 | 140G1 | 140G5 | 140G6 | 140G11 | 140H1 | 140H4 |
| 1 | BG8743 | 23 | 1 | 1 | No | **-** | **Y** | **Y** | **-** | **-** | **Y** | **Y** | **Y** | **Y** | **Y** | **Y** |
| 2 | AC094 | 9L | 1 | 1 | Yes | **Y** | **Y** | **Y** | **Y** | **Y** | **Y** | **Y** | **Y** | **Y** | **Y** | **Y** |
| 3 | BG6692 | 33 | 1 | 1 | Yes | **Y** | **Y** | **Y** | **Y** | **Y** | **Y** | **Y** | **Y** | **Y** | **Y** | **Y** |
| 4 | BG8838 | 6 | 1 | 1 | Yes | **Y** | **Y** | **Y** | **Y** | **Y** | **Y** | **Y** | **Y** | **Y** | **Y** | **Y** |
| 5 | DBL1 | 6B | 1 | 1 | Yes | **Y** | **Y** | **Y** | **Y** | **Y** | **Y** | **Y** | **Y** | **Y** | **Y** | **Y** |
| 6 | BG9739 | 4 | 1 | 1 | No | **-** | **-** | **Y** | **Y** | **Y** | **Y** | **Y** | **Y** | **Y** | **Y** | **Y** |
| 7 | DBL6A | 6A | 1 | 1 | Yes | **Y** | **Y** | **Y** | **Y** | **Y** | **Y** | **Y** | **Y** | **Y** | **Y** | **Y** |
| 8 | L81905 | 4 | 1 | 1 | No | **-** | **-** | **Y** | **Y** | **Y** | **Y** | **Y** | **Y** | **Y** | **Y** | **Y** |
| 9 | DBL5 | 5 | 1 | 2 | No | **-** | **-** | **Y** | **Y** | **Y** | **Y** | **Y** | **Y** | **Y** | **Y** | **Y** |
| 10 | E134 | 23F | 1 | 2 | No | **-** | **-** | **-** | **-** | **-** | **-** | **-** | **-** | **-** | **Y** | **-** |
| 11 | EF10197 | 3 | 1 | 2 | Yes | **-** | **-** | **Y** | **Y** | **Y** | **Y** | **Y** | **Y** | **Y** | **Y** | **Y** |
| 12 | EF6796 | 6A | 1 | 2 | No | **-** | **-** | **-** | **-** | **-** | **-** | **-** | **-** | **-** | **Y** | **-** |
| 13 | BG9163 | 6B | 1 | 2 | No | **-** | **-** | **-** | **-** | **-** | **-** | **-** | **-** | **-** | **Y** | **-** |
| 14 | AC122 | 9V | 2 | 3 | No | **Y** | **Y** | **Y** | **Y** | **Y** | **Y** | **Y** | **Y** | **Y** | **Y** | **Y** |
| 15 | BG8090 | 19 | 2 | 3 | No | **Y** | **Y** | **Y** | **Y** | **Y** | **Y** | **Y** | **Y** | **Y** | **Y** | **Y** |
| 16 | BG7561 | 15 | 2 | 4 | Yes | **Y** | **Y** | **Y** | **Y** | **Y** | **Y** | **Y** | **Y** | **Y** | **Y** | **Y** |
| 17 | BG7817 | 12 | 2 | 4 | Yes | **Y** | **Y** | **Y** | **Y** | **Y** | **Y** | **Y** | **Y** | **Y** | **Y** | **Y** |
| 18 | BG11703 | 18 | 2 | 4 | Yes | **Y** | **Y** | **Y** | **Y** | **Y** | **Y** | **Y** | **Y** | **Y** | **Y** | **Y** |
| 19 | EF5668 | 4 | 2 | 4 | Yes | **-** | **-** | **Y** | **Y** | **Y** | **Y** | **Y** | **Y** | **Y** | **Y** | **Y** |
| 20 | BG6380 | 37 | 3 | 6 | Yes | **-** | **-** | **Y** | **Y** | **Y** | **Y** | **Y** | **Y** | **Y** | **Y** | **Y** |
|  |  | Number of strains bound | | | | 10/20 | 11/20 | 17/20 | 16/20 | 16/20 | 17/20 | 17/20 | 17/20 | 17/20 | 20/20 | 17/20 |
|  |  | Overall binding (%) | | | | 50% | 55% | 85% | 80% | 80% | 85% | 85% | 85% | 85% | 100% | 85% |
