# Supplementary material for: Generation and Improvement of Effector Function of a Novel Broadly Reactive and Protective Monoclonal Antibody against Pneumococcal Surface Protein A of Streptococcus pneumoniae
Source: PLoS One. 2016 May 12;11(5):e0154616. doi: 10.1371/journal.pone.0154616 (PMC4865217; doi:10.1371/journal.pone.0154616)
Supplement: S2 Table — Affinity of lead anti-PspA mAbs (139G3, 140G1, 140G11, and 140H1) to recombinant PspA was measured by surface plasmon resonance (SPR) with a Biacore 3000 (GE Healthcare Bio-Sciences) using sensor chips with immobilized PspA ligand. Bivalent binding modeling was used to analyze the binding affinity of each mAb (Biacore 3000 Evaluation software, Biacore). (DOCX) [file pone.0154616.s003.docx]

**S2 Table: Biacore-based evaluation of affinity of lead anti-PspA mouse antibodies to recombinant PspA-D39, PspA-BAA-658, and PspA-TIGR4.**

| Ligand: PspA-D39 (~160 RUs); Analyte: 140H1 (Concentration Range = 40nM-1.25nM) | | | | | | | |
| --- | --- | --- | --- | --- | --- | --- | --- |
| **ka1 (1/Ms)** | **kd1 (1/s)** | **KD1** | **ka2 (1/RUs)** | **kd2 (1/s)** | **KD2** | **Rmax (RU)** | **Chi2** |
| 1.22E+05 | 4.29E-04 | 3.52E-09 | 8.74E-03 | 0.244 | 2.79E+01 | 247 | 1.61 |
| Ligand: PspA-D39 (~160 RUs); Analyte: 140G1 (Concentration Range = 40nM-1.25nM) | | | | | | | |
| **ka1 (1/Ms)** | **kd1 (1/s)** | **KD1** | **ka2 (1/RUs)** | **kd2 (1/s)** | **KD2** | **Rmax (RU)** | **Chi2** |
| 6.26E+05 | 1.49E-03 | 2.38E-09 | 0.0471 | 0.289 | 6.136 | 349 | 4.4 |
| Ligand: PspA-D39 (~160 RUs); Analyte: 140G11 (Concentration Range = 40nM-1.25nM) | | | | | | | |
| **ka1 (1/Ms)** | **kd1 (1/s)** | **KD1** | **ka2 (1/RUs)** | **kd2 (1/s)** | **KD2** | **Rmax (RU)** | **Chi2** |
| 9.58E+05 | 3.22E-03 | 3.36E-09 | 0.18 | 0.511 | 2.839 | 357 | 8.03 |
| Ligand: PspA-D39 (~160 RUs); Analyte: 139G3 (Concentration Range = 40nM-1.25nM) | | | | | | | |
| **ka1 (1/Ms)** | **kd1 (1/s)** | **KD1** | **ka2 (1/RUs)** | **kd2 (1/s)** | **KD2** | **Rmax (RU)** | **Chi2** |
| 1.70E+04 | 0.0121 | 7.12E-07 | 1.48E-05 | 3.11E-03 | 2.10E+02 | 35.7 | 0.0493 |
| Ligand: PspA-BAA-658 (~136 RUs); Analyte: 140H1 (Concentration Range = 40nM-1.25nM) | | | | | | | |
| **ka1 (1/Ms)** | **kd1 (1/s)** | **KD1** | **ka2 (1/RUs)** | **kd2 (1/s)** | **KD2** | **Rmax (RU)** | **Chi2** |
| 2.18E+05 | 1.75E-04 | 8.03E-10 | 2.97E-03 | 0.128 | 4.31E+01 | 241 | 2.02 |
| Ligand: PspA-BAA-658 (~136 RUs); Analyte: 140G1 (Concentration Range = 40nM-1.25nM) | | | | | | | |
| **ka1 (1/Ms)** | **kd1 (1/s)** | **KD1** | **ka2 (1/RUs)** | **kd2 (1/s)** | **KD2** | **Rmax (RU)** | **Chi2** |
| 7.05E+05 | 5.53E-03 | 7.84E-09 | 105 | 450 | 4.29E+00 | 307 | 3.5 |
| Ligand: PspA-BAA-658 (~136 RUs); Analyte: 140G11 (Concentration Range = 40nM-1.25nM) | | | | | | | |
| **ka1 (1/Ms)** | **kd1 (1/s)** | **KD1** | **ka2 (1/RUs)** | **kd2 (1/s)** | **KD2** | **Rmax (RU)** | **Chi2** |
| 1.26E+06 | 2.75E-02 | 2.18E-08 | 0.0188 | 0.28 | 1.49E+01 | 226 | 3.06 |
| Ligand: PspA-BAA-658 (~136 RUs); Analyte: 139G3 (Concentration Range = 40nM-1.25nM) | | | | | | | |
| **ka1 (1/Ms)** | **kd1 (1/s)** | **KD1** | **ka2 (1/RUs)** | **kd2 (1/s)** | **KD2** | **Rmax (RU)** | **Chi2** |
| 1.83E+05 | 1.67E-03 | 9.13E-09 | 0.00832 | 0.415 | 4.99E+01 | 158 | 0.637 |
| Ligand: PspA-TIGR4 (~106 RUs); Analyte: 140H1 (Concentration Range = 20nM-0.625nM) | | | | | | | |
| **ka1 (1/Ms)** | **kd1 (1/s)** | **KD1** | **ka2 (1/RUs)** | **kd2 (1/s)** | **KD2** | **Rmax (RU)** | **Chi2** |
| 3.90E+05 | 3.23E-02 | 8.28E-08 | 3.41E-02 | 0.193 | 5.66E+00 | 25.1 | 0.655 |
| Ligand: PspA-TIGR4 (~106 RUs); Analyte: 140G1 (Concentration Range = 20nM-0.625nM) | | | | | | | |
| **ka1 (1/Ms)** | **kd1 (1/s)** | **KD1** | **ka2 (1/RUs)** | **kd2 (1/s)** | **KD2** | **Rmax (RU)** | **Chi2** |
| N/A | N/A | N/A | N/A | N/A | N/A | N/A | N/A |
| Ligand: PspA-TIGR4 (~106 RUs); Analyte: 140G11 (Concentration Range = 20nM-0.625nM) | | | | | | | |
| **ka1 (1/Ms)** | **kd1 (1/s)** | **KD1** | **ka2 (1/RUs)** | **kd2 (1/s)** | **KD2** | **Rmax (RU)** | **Chi2** |
| N/A | N/A | N/A | N/A | N/A | N/A | N/A | N/A |
| Ligand: PspA-TIGR4 (~106 RUs); Analyte: 139G3 (Concentration Range = 20nM-0.625nM) | | | | | | | |
| **ka1 (1/Ms)** | **kd1 (1/s)** | **KD1** | **ka2 (1/RUs)** | **kd2 (1/s)** | **KD2** | **Rmax (RU)** | **Chi2** |
| 1.85E+05 | 0.000186 | 1.01E-09 | 5.28E-04 | 5.12E-02 | 9.70E+01 | 313 | 0.426 |
