# Supplementary material for: Generation and Improvement of Effector Function of a Novel Broadly Reactive and Protective Monoclonal Antibody against Pneumococcal Surface Protein A of Streptococcus pneumoniae
Source: PLoS One. 2016 May 12;11(5):e0154616. doi: 10.1371/journal.pone.0154616 (PMC4865217; doi:10.1371/journal.pone.0154616)
Supplement: S3 Table — The indicated pneumococcal strains were grown to exponential phase in THY, washed in HBSS, 5% BSA, then incubated in 0.2 mL HBSS, 3.75% BSA at 37°C with 600 rpm shaking with or without isotype control or anti-PspA mAbs (IgG2a) and commercially available mouse serum at concentrations that were optimized for each pneumococcal strain. After 30 min, cells were washed with ice-cold PBS, 0.5% BSA, and then resuspended in 100 μL PBS, 0.5% BSA containing 2 μg/mL fluorescein-labeled anti-mouse C3 antibody. After 30–60 min incubation at 4°C without mixing, bacteria were washed in ice-cold PBS, 0.5% BSA. Cells were fixed and subjected to flow cytometry. +++, strong C3 deposition; ++, moderate C3 deposition; +, weak C3 deposition; +/-, barely detectable activity; -, no detectable activity; n.a., not tested, since antibody does not bind to respective S. pneumoniae strain. All scoring was done using binding of negative control antibody as a comparator. (DOCX) [file pone.0154616.s004.docx]

**S3 Table. Anti-PspA mAbs show strain specific activity in complement deposition assays (CDAs) with pneumococcal strains representing PspA clades 1-5.**

|  | ***S. pneumoniae* strains tested** | | | | **Detection of C3b deposition on bacteria** | | | |
| --- | --- | --- | --- | --- | --- | --- | --- | --- |
| **#** | **Strain** | **Serotype** | **PspA Family** | **PspA Clade** | **139G3** | **140csG1** | **140G11** | **140csH1** |
| **1** | **ATCC-49619** | **19F** | **1** | **1** | **+++** | **+++** | **+++** | **+++** |
| **2** | **BAA-658** | **6B** | **1** | **1** | **+++** | **+++** | **+++** | **+++** |
| **3** | **ATCC-6305** | **5** | **1** | **2** | **n.a.** | **+/-** | **+/-** | **++** |
| **4** | **WU2** | **3** | **1** | **2** | **n.a.** | **+++** | **+++** | **++** |
| **5** | **D39** | **2** | **1** | **2** | **n.a.** | **+++** | **+++** | **+++** |
| **6** | **PJ-1324** | **6B** | **2** | **3** | **+++** | **+++** | **+++** | **+++** |
| **7** | **TIGR4** | **4** | **2** | **3** | **+++** | **+** | **+** | **+++** |
| **8** | **NCTC-11905** | **18C** | **2** | **4** | **+++** | **+++** | **+++** | **+++** |
| **9** | **ATCC-700673** | **6B** | **2** | **5** | **++** | **++** | **++** | **++** |
