# Supplementary material for: Generation and Improvement of Effector Function of a Novel Broadly Reactive and Protective Monoclonal Antibody against Pneumococcal Surface Protein A of Streptococcus pneumoniae
Source: PLoS One. 2016 May 12;11(5):e0154616. doi: 10.1371/journal.pone.0154616 (PMC4865217; doi:10.1371/journal.pone.0154616)
Supplement: S4 Table — 4–6 h before i.p., i.n., or i.v. infection with the indicated S. pneumoniae strains, mice were pretreated i.p. with the indicated amounts of mAbs in PBS. In most experiments, heparinized tail vein blood was collected and bacterial CFU enumerated. Survival was monitored for 13–15 days. The combined survival results of 1–3 independent experiments are shown for each strain. *, p<0.05; **, p<0.005; ***, p<0.0005, survival curve, isotype control vs. anti-PspA antibodies with Mantel-Cox test; CFU, no significant protection in terms of survival, but significantly reduced bacterial numbers in tail vein blood observed 24 h after infection compared to negative control antibody treated mice; n.t., not tested. Please note that CD-1 mice were used for all but the WU2 passive immunization model in which Swiss Webster mice were used. (DOCX) [file pone.0154616.s005.docx]

**S4 Table. Activity of anti-PspA mAbs in mouse passive immunization models of septicemia with pneumococcal strains representating PspA clades 1-5.**

| ***S. pneumoniae* strains used for mouse passive immunization experiments, infection routes, CFU and antibody doses** | | | | | | | | **Mice alive 13-15 days after infection** | | | | |
| --- | --- | --- | --- | --- | --- | --- | --- | --- | --- | --- | --- | --- |
| **#** | **Strain** | **Serotype** | **PspA Family** | **PspA**  **Clade** | **Infection route** | **CFU dose** | **mAb dose (μg)** | **Isotype mAb C44** | **139G3** | **140csG1** | **140G11** | **140csH1** |
| **1** | **ATCC-6301** | **1** | **1** | **1** | **i.p.** | **4.6-8.3x10^6^** | **250** | **2/20** | **6/15^*^** | **3/15^n.s.^** | **3/15^n.s.^** | **10/25^*^** |
| **2** | **BAA-658** | **6B** | **1** | **1** | **i.p.** | **0.6-1.0x10^8^** | **250** | **0/15** | **10/15^***^** | **6/15^***^** | **2/14^*^** | **3/14^*^** |
| **3** | **ATCC-49619** | **19F** | **1** | **1** | **i.p.** | **2.0-2.7x10^8^** | **150-300** | **0/14** | **0/4^n.s.^** | **0/9^n.s.^** | **0/4^n.s.^** | **0/9 ^n.s.^** |
| **4** | **D39** | **2** | **1** | **2** | **i.p.** | **4.0x10^2^** | **300** | **0/10** | **n.t.** | **5/10^*^** | **5/10^*^** | **4/10^*^** |
| **5** | **NCTC-7978** | **3** | **1** | **2** | **i.p.** | **1.0x10^2^** | **250** | **0/10** | **n.t.** | **0/7^*^** | **0/7^*^** | **n.t.** |
| **6** | **WU2** | **3** | **1** | **2** | **i.n.** | **1.5x10^7^** | **300** | **0/11** | **n.t.** | **9/12^***^** | **5/11^***^** | **6/12^***^** |
| **7** | **ATCC-6305** | **5** | **1** | **2** | **i.v.** | **0.9-1.7x10^4^** | **200** | **0/10** | **n.t.** | **0/5 ^n.s.^** | **0/5 ^n.s.^** | **0/5 ^n.s.^** |
| **8** | **NCTC-11886** | **4** | **2** | **3** | **i.p.** | **3.0x10^2^** | **250** | **0/15** | **4/15^*^** | **5/15^*^** | **4/15^*^** | **4/15^*^** |
| **9** | **TIGR4** | **4** | **2** | **3** | **i.p.** | **1.6-2.0x10^7^** | **250** | **0/10** | **4/10^*^** | **1/20^n.s.^** | **0/10^n.s.^** | **2/20^*^** |
| **10** | **PJ-1324** | **6B** | **2** | **3** | **i.p.** | **1.0-1.4x10^3^** | **100** | **0/8** | **8/8^***^** | **6/8^***^** | **4/8^***^** | **8/8^***^** |
| **11** | **NCTC-11905** | **18C** | **2** | **4** | **i.p.** | **8.0x10^7^** | **150-200** | **0/15** | **5/10^*^** | **7/10^***^** | **4/10^*^** | **3/10^*^** |
| **12** | **ATCC-6303** | **3** | **2** | **5** | **i.v.** | **6.0-7.5x10^5^** | **200** | **0/10** | **7/10^***^** | **7/10^***^** | **1/10^n.s.^** | **n.t.** |
| **13** | **BAA-612** | **6B** | **2** | **5** | **i.p.** | **8.0x10^5^** | **250** | **0/10** | **0/10^CFU^** | **1/10^n.s.^** | **0/10^n.s.^** | **2/10^CFU^** |
|  |  |  |  |  |  |  |  |  | **8/9 (89%)** | **8/13**  **(62%)** | **7/13**  **(54%)** | **9/11**  **(82%)** |
|  |  |  |  |  |  |  |  |  | **Total of models in which anti-PspA mAb activity was observed (%)** | | | |
